# An Optimistic Solver for the Mathematical Model of the Flow of Johnson Segalman Fluid on the Surface of an Infinitely Long Vertical Cylinder

**DOI:** 10.3390/ma14247798

**Published:** 2021-12-16

**Authors:** Naveed Ahmad Khan, Fahad Sameer Alshammari, Carlos Andrés Tavera Romero, Muhammad Sulaiman, Seyedali Mirjalili

**Affiliations:** 1 Department of Mathematics, Abdul Wali Khan University, Mardan 23200, Pakistan; ahmednaveed854477@gmail.com (N.A.K.); msulaiman@awkum.edu.pk (M.S.); 2 Department of Mathematics, College of Science and Humanities in Alkharj, Prince Sattam bin Abdulaziz University, Al-Kharj 11942, Saudi Arabia; 3 COMBA R&D Laboratory, Faculty of Engineering, Universidad Santiago de Cali, Cali 76001, Colombia; carlos.tavera00@usc.edu.co; 4 Centre for Artificial Intelligence Research and Optimisation, Torrens University Australia, Fortitude Valley, Brisbane, QLD 4006, Australia; ali.mirjalili@gmail.com; 5 Yonsei Frontier Lab, Yonsei University, Seoul 03722, Korea

**Keywords:** drainage problems, Johnson Segalman model, computational fluid dynamics, weighted legendre neural networks, hybrid soft computing, generalized normal distribution optimization, sequential quadratic programming

## Abstract

In this paper, a novel soft computing technique is designed to analyze the mathematical model of the steady thin film flow of Johnson–Segalman fluid on the surface of an infinitely long vertical cylinder used in the drainage system by using artificial neural networks (ANNs). The approximate series solutions are constructed by Legendre polynomials and a Legendre polynomial-based artificial neural networks architecture (LNN) to approximate solutions for drainage problems. The training of designed neurons in an LNN structure is carried out by a hybridizing generalized normal distribution optimization (GNDO) algorithm and sequential quadratic programming (SQP). To investigate the capabilities of the proposed LNN-GNDO-SQP algorithm, the effect of variations in various non-Newtonian parameters like Stokes number ($S_{t}$), Weissenberg number ($W_{e}$), slip parameters (*a*), and the ratio of viscosities (ϕ) on velocity profiles of the of steady thin film flow of non-Newtonian Johnson–Segalman fluid are investigated. The results establish that the velocity profile is directly affected by increasing Stokes and Weissenberg numbers while the ratio of viscosities and slip parameter inversely affects the fluid’s velocity profile. To validate the proposed technique’s efficiency, solutions and absolute errors are compared with reference solutions calculated by RK-4 (ode45) and the Genetic algorithm-Active set algorithm (GA-ASA). To study the stability, efficiency and accuracy of the LNN-GNDO-SQP algorithm, extensive graphical and statistical analyses are conducted based on absolute errors, mean, median, standard deviation, mean absolute deviation, Theil’s inequality coefficient (TIC), and error in Nash Sutcliffe efficiency (ENSE). Statistics of the performance indicators are approaching zero, which dictates the proposed algorithm’s worth and reliability.

## 1. Introduction

In recent times, the importance of non-Newtonian fluids has become prominent with developments in industries like pulp, petroleum and polymer and so forth. Many industrial applications, such as the melting of polymers, asphalts, biological solutions, paints and glues, fall into this category. Due to the complex nature of non-Newtonian fluids, it is difficult to establish a single mathematical model that can describe all properties of the fluid. As a result, several fluid models have been presented to predict non-Newtonian behaviour of various materials. The Generalized third grade fluid model has received significant attention among these [1]. Denson [2], N. V. Lavrik [3], Tasawar Hayat [4] studied various third grade fluid models. M.M Bhatti [5] studied the sinusoidal motion of small particles through a Darcy–Brinkman–Forchheimer microchannel filled with non-Newtonian fluid under electro-osmotic forces. Landau and Lifshitz [6] for the first time discuss thin-film flow of non-Newtonian Johnson–Segalman fluid. Siddiquie [7] found analytical results for drainage problem of fourth graded fluid over a vertical cylinder and also calculated exact solutions for Phan Thein Tanner (PTT) fluid for lifting and drainage problems [8]. Alam [9] studied thin-film flow of Johnson Segalman fluid on vertical surfaces.

Various models of fluids explain the non-Newtonian behavior of fluids. However, Johnson–Segalman fluid has gained many researchers’ interest because it includes exceptional cases of classical Newtonian fluid like Oldroyd B fluid and Maxwell fluid [10]. The Johnson–Segalman fluid model is considered a viscoelastic model developed to allow non-affine deformations [11]. Many researchers discuss spurt phenomena of this non Newtonian fluid model [12,13,14]. Rao [15] investigated the flow of Johnson–Segalman fluid model with and without suction on rotating coaxial cylinders. Rajagopal [16] studied three different cylindrical Poiseuille flows of Johnson–Segalman fluid. Unlike most other fluid models, Johnson–Segalman fluid allows a nonmonotonic relationship between the rate of share and shear stress in a simple shear flow for different material parameters. T. Hayat [17] used the concept of this model for peristaltic flow.

Most of the fluid models, are governed by partial and ordinary differential equations and generally, finding a solution to such a models is always a challenging task. Theoretical researchers in mathematics have developed various methods to find analytical expressions and approximate solutions with proven convergence for nonlinear differential equations. Adomian decomposition methods (ADM) [18,19], Variational iteration method (VIM) [20], Finite difference method (FDM) [21,22] and Optimal homotopy perturbation method (OHAM) [23,24] are used to solve variety of ordinary and partial differential equation models. GM Sobamowo [25] used Galerkin’s weighted residuals method to present an approximate solution for heat transfer in a pipe with Johnson-Segalman fluid. The motion of Johnson–Segalman fluid in an inclined channel subject to radiative flux was studied by Hayat [26] using the perturbation method. In terms of consistency, convergence, robustness, and applicability, all of these implemented techniques have their advantages and limitations. These methods are based on well established deterministic procedures. On the other hand, soft computing techniques based on neural networks are relatively less exploited and rapidly convergent in obtaining a solution to non-linear differential equations. In the recent past, numerical solutions based on artificial intelligence (AI) through neural networks, optimized with bio/nature-inspired global and local optimization techniques have gained the research community’s attention. Raja [27] designed a bio-inspired approach using the hybridization of genetic algorithm and an active set algorithm (GA-ASA) to solve Johnson’s steady thin-film flow Segalman fluid through vertical cylinder for drainage problems. N.A Khan [28] used Legendre neural networks (LeNN) and optimized the model of counter-current imbibition phenomena during the secondary oil recovery process by using the nature-inspired whale optimization algorithm and the Nelder–Mead algorithm. Some recent applications of stochastic computational techniques or learning algorithms based on artificial neural networks (ANN’s) with novel metaheuristic and heuristic techniques includes the solution of wire coating dynamics with Oldroyd 8-constant fluid [29,30], nonlinear problems arising in heat transfer [31,32], absorption of CO2 into solution of phenyl glycidyl ether (PGE) [33], mathematical models of CBSC over wireless channels [34] and electrohydrodynamic (EHD) flow in a circular cylindrical conduit [35]. Recent developments in stochastic algorithms for such problems motivated authors to explore and exploit machine learning algorithms and use Legendre neural networks to develop an alternative, accurate, and reliable framework to solve nonlinear multi-singular initial or boundary value problems representing drainage problems.

Salient features of the presented study are summarized as follows:

- The Mathematical formulation for non Newtonian Johnson–Segalman fluid is presented using the law of conservation of mass and momentum under sufficient boundary conditions that result in partial differential equations. The drainage problem is further reduced to non-linear ordinary differential equation employing similarity transformation;
- This study aims to introduce a novel solution computing that involves Legendre artificial neural networks and two algorithms: generalized normal distribution optimization (GNDO) and sequential quadratic programming (SQP). GNDO is used as a global search technique while SQP is utilized as a local search algorithm;
- Effect of variations in different parameters like Weissenberg number ($W_{e}$), Stokes number ($S_{t}$), slip parameter (*a*) and the ratio of viscosities (ϕ) on velocity profile of steady thin film flow of non-Newtonian Johnson–Segalman fluid is investigated.
- Performance indicators are used for different cases of drainage problem studied in this paper to validate the efficiency and correctness of the LNN-GNDO-SQP algorithm;
- Extensive statistical and graphical analysis in terms of absolute errors, fitness evaluation, MAD, RMSE, TIC, and ENSE are provided, that demonstrated the ability of our proposed algorithm in solving real-world problems.

## 2. Mathematical Formulation of Drainage Problem

In this section, the mathematical formulation of non-Newtonian Johnson–Segalman fluid on the outer surface of a long vertical cylinder for a drainage problem is briefly discussed [9].

### 2.1. Basic Equations

Basic equations governing the flow of incompressible fluid neglecting the thermal effects are given as:

(1) $$\nabla .\underline{V}=0,$$

(2) $$\rho \frac{D\underline{V}}{Dt}=\nabla .\sigma +\rho f,$$

where $\underline{V}$ is velocity vector of fluid, f is body force, $\frac{D}{Dt}$ is material time derivative, ρ denotes density, Cauchy stress tensor is presented by σ and in case of Johnson–Segalman fluid, it is defined by [10]:

(3) σ=T−pI,

(4) T=S+2μD,

(5) $$S+m\left[\frac{DS}{Dt}+S\left(W-aD\right)+{\left(W-aD\right)}^{T}S\right]=2\eta D,$$

(6) $$\frac{DS}{Dt}=\frac{\partial S}{\partial t}+\left(gradS\right)V,$$

in Equation (3), −pI is an intermediate part of stress due to incompressibility, *a* is slip parameter and viscosities are denoted by μ and η.

(7) $$D=\frac{1}{2}\left[L+L^{T}\right],\;W=\frac{1}{2}\left[L-L^{T}\right],$$

where D and W are symmetric and skew symmetric components of velocity gradient respectively. L which is defined as $grad\underline{V}$. In case of η=μ=0 and a=1, non-Newtonian Johnson–Segalman fluid is transformed into the newtonian model and Maxwell fluid respectively.

### 2.2. Formulation

**Figure 1:**
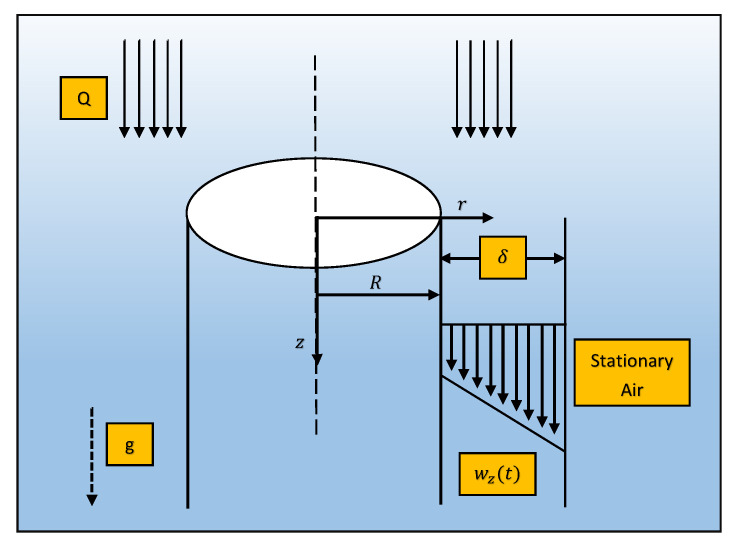
Schematic view of drainage problem.

A vertically long cylinder of radius *R* is considered with non-Newtonian Johnson–Segalman fluid on its outer surface as shown in Figure 1. The fluid is considered in the form of uniform axisymmetric thin film with thickness δ and in stationary contact with air. It is assumed that time has no effect on flow (steady state), surface tension is zero and the pressure acting on fluid is atmospheric pressure, so the velocity profile $\underline{V}$ is given as

(8) $$\underline{V}=\left[0,0,w(r)\right].$$

Radial direction is considered parpendicular to cylinder while z-axis is in downward direction horizontal to cylinder as prescribed in Figure 1. Boundary conditions for free space are given as:

(9) $$\;\mathrm{at}\;r=R+\delta ,\;T_{rz}=0,$$

In case of no slip conditions, we have:

(10) atr=R,w=0;

here, $T_{rz}$ denotes component of shear stress. Continuity equation Equation (1) is satisfied identically by using Equations (3) and (4) thus Equation (2) is reduce to

(11) $$0=-\frac{\partial p}{\partial r}+\rho f_{1},$$

(12) $$0=-\frac{1}{r}\frac{\partial p}{\partial \theta }+\rho f_{2},$$

(13) $$0=-\frac{\partial p}{\partial z}+\frac{1}{r}\frac{d\left(rT_{rz}\right)}{dr}+\rho f_{3},$$

components of force in polar coordinates *r*, θ and *z* components are presented by $f_{1}$, $f_{2}$ and $f_{3}$ respectively. Since pressure is assumed to be constant and gravitational force is acting vertically downward, therefore Equation (13) can be written as:

(14) $$0=\frac{1}{r}\frac{d\left(rT_{xy}\right)}{dr}+\rho g.$$

The non-zero component of S is obtained by using Equations (6) and (7); in Equation (5), we get:

(15) $$S_{rr}=\frac{\left(a-1\right)\eta m{\left(\frac{dw}{dr}\right)}^{2}}{1-\left(a^{2}-1\right)m^{2}{\left(\frac{dw}{dr}\right)}^{2}},$$

(16) $$S_{rz}=S_{zr}=\frac{\eta \left(\frac{dw}{dr}\right)}{1+m^{2}\left(1-a^{2}\right){\left(\frac{dw}{dr}\right)}^{2}},$$

(17) $$S_{zz}=\frac{\eta m\left(1+a\right){\left(\frac{dw}{dr}\right)}^{2}}{1-\left(a^{2}-1\right)m^{2}{\left(\frac{dw}{dr}\right)}^{2}}.$$

By using Equations (15)–(17) in Equation (4), the following components of Cauchy stress tensor T are obtained:

(18) $$T_{rr}=\frac{\left(a-1\right)\eta m{\left(\frac{dw}{dr}\right)}^{2}}{1-\left(a^{2}-1\right)m^{2}{\left(\frac{dw}{dr}\right)}^{2}},$$

(19) $$T_{rz}=T_{zr}=\mu \left(\frac{dw}{dr}\right)+\frac{\eta \left(\frac{dv}{dr}\right)}{1-\left(a^{2}-1\right)m^{2}{\left(\frac{dw}{dr}\right)}^{2}},$$

(20) $$T_{zz}=\frac{\eta m\left(1+a\right){\left(\frac{dw}{dr}\right)}^{2}}{1+m^{2}\left(1-a^{2}\right){\left(\frac{dw}{dr}\right)}^{2}}.$$

Now, Equation (13) can be written as:

(21) $$\frac{d}{dr}\left[r\left(\mu \left(\frac{dw}{dr}\right)+\frac{\eta \left(\frac{dw}{dr}\right)}{1+m^{2}\left(1-a^{2}\right){\left(\frac{dw}{dr}\right)}^{2}}\right)\right]=-\rho gr,$$

with boundary conditions

(22) $$\;\mathrm{at}\;r=R+\delta ,\;\frac{dw}{dr}=0\;\;(\mathrm{free}\;\mathrm{surface}),$$

(23) atr=R,w=0(noslipcondition).

Now, using similarity transformation by defining the following dimensionless parameters,

(24) $$w^{*}=\frac{w}{U_{0}},\;r^{*}=\frac{r}{\delta },\;\delta^{*}=\frac{\delta }{R},\;T_{rz}^{*}=\frac{T_{rz}}{\left(\mu +\eta \right)\frac{U_{0}}{\delta }},\;\phi =\frac{\mu }{\left(\mu +\eta \right)},$$

using the equations in (24) and omitting * in Equation (21), we have:

(25) $$\frac{d}{dr}\left[r\left(\phi \left(\frac{dw}{dr}\right)+\frac{\left(1-\phi \right)\left(\frac{dw}{dr}\right)}{1+W_{e}^{2}\left(1-a^{2}\right){\left(\frac{dw}{dr}\right)}^{2}}\right)\right]=-rS_{t},$$

with conditions

(26) $$\;\mathrm{at}\;r=R+\delta ,\;\frac{dw}{dr}=0\;\;(\mathrm{free}\;\mathrm{surface})\;,$$

(27) atr=1,w=0(noslipcondition),

where $W_{e}$ and $S_{t}$ are Weissenberg and Stokes numbers respectively and defined are as

(28) $$W_{e}=\frac{mU_{o}}{\delta },\;S_{t}=\frac{\rho g\delta^{2}}{\mu_{eff}U_{0}}\;\mathrm{and}\;\mu_{eff}=\left(\eta +\mu \right).$$

Integration of Equation (25) yields the drainage problem as under

(29) $$\begin{array}{cc} \frac{dw}{dr} & +\phi W_{e}^{2}\left(1-a^{2}\right){\left(\frac{dw}{dr}\right)}^{3}-\frac{S_{t}}{2}W_{e}^{2}\left(1-a^{2}\right)\left({\left(1+\delta \right)}^{2}\frac{1}{r}-r\right){\left(\frac{dw}{dr}\right)}^{2}\\ \; & =\frac{S_{t}}{2}\left({\left(1+\delta \right)}^{2}\frac{1}{r}-r\right),\;w\left(1\right)=0, \end{array}$$

w(r) denotes the velocity profile of Johnson–Segalman fluid in the radial direction.

## 3. The LNN-GNDO-SQP Algorithm

The designed scheme consists of two major parts; first, a Legendre polynomials-based artificial neural networks (LNNs) model is developed and, secondly, the neurons in LNN architecture for the drainage problem are optimized by using hybridization of GNDO and SQP algorithms.

### 3.1. Series Solution Based on LNN Structure

A mathematical model of an approximate solution for the drainage problem is developed by using an LNN structure in terms of weighted Legendre polynomials. A trial or approximate series solution w(r) with first order derivative $w^{\prime }\left(r\right)$ in terms of layers in LNN is mathematically expressed as:

(30) $$w\left(r\right)=\sum_{j=1}^{m}\alpha_{j}L_{n}\left(\omega_{j}r+\beta_{j}\right),$$

where $\alpha_{i}$, $\omega_{i}$ and $\beta_{i}$ are design weights and *n* shows the number of Legendre polynomials involved in Equation (30) and

(31) $$\frac{dw}{dr}\left(r\right)=\sum_{j=1}^{m}\alpha_{j}\frac{dL_{n}}{dr}\left(\omega_{j}r+\beta_{j}\right),$$

where $L_{n}$ denotes Legendre polynomials. The first three Legendre polynomials are given as:

(32) $$L_{1}\left(r\right)=1,\;L_{2}\left(r\right)=r,\;L_{3}\left(r\right)=\frac{1}{2}\left(3r^{2}-1\right),\;L_{4}\left(r\right)=\frac{1}{2}\left(5r^{3}-3r\right).$$

Higher order polynomials are generated by:

(33) $$L_{n+1}\left(r\right)=\frac{1}{n+1}\left[\left(2n+1\right)rL_{n}\left(r\right)-nL_{n-1}\left(r\right)\right].$$

**Figure 2:**
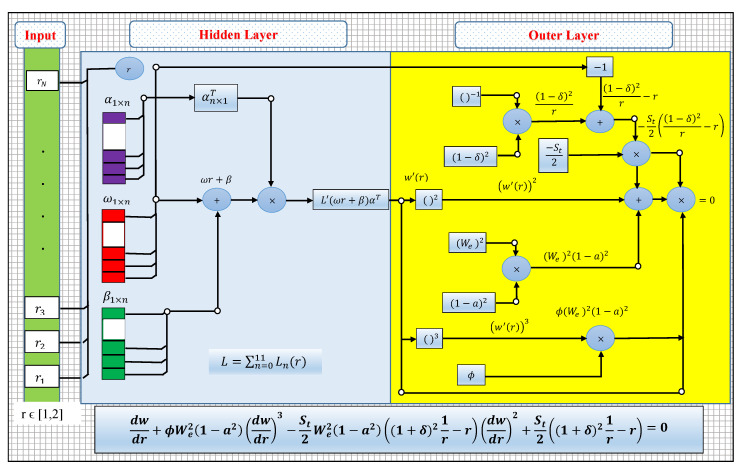
Legendre polynomial based artificial neural networks architecture for drainage problem.

The ANN structure for the drainage problem in terms of the input and hidden and outer layer is shown in Figure 2.

### 3.2. Construction of Fitness Function

An objective function also known as a fitness function or merit function is constructed based on mean square errors in candidate solutions to train unknown weights in LNN. Structure of fitness function is formulated as:

(34) $$\min\;\varepsilon =\varepsilon_{1}+\varepsilon_{2},$$

$\varepsilon_{1}$ is mean square error in candidate solution by using it in the drainage problem which is:

(35) $$\varepsilon_{1}=\frac{1}{n}\sum_{m=1}^{n}{\left(\begin{array}{c} \frac{d{\hat{w}}_{m}}{dr}+\phi W_{e}^{2}\left(1-a^{2}\right){\left(\frac{d{\hat{w}}_{m}}{dr}\right)}^{3}-\frac{S_{t}}{2}W_{e}^{2}\left(1-a^{2}\right)\\ \times \left({\left(1+\delta \right)}^{2}\frac{1}{r}-r\right){\left(\frac{d{\hat{w}}_{m}}{dr}\right)}^{2}-\frac{S_{t}}{2}\left({\left(1+\delta \right)}^{2}\frac{1}{r}-r\right) \end{array}\right)}^{2},$$

where $N=\frac{1}{h},{\hat{w}}_{m}=\hat{w}\left(r_{m}\right),r_{m}=mh$ $\varepsilon_{2}$ is the mean square error in the candidate solution by putting it in the initial condition, which is

(36) $$\varepsilon_{2}={\left(w\left(1\right)-0\right)}^{2}.$$

### 3.3. Optimization Framework Used to Compute Best Weights

**Figure 3:**
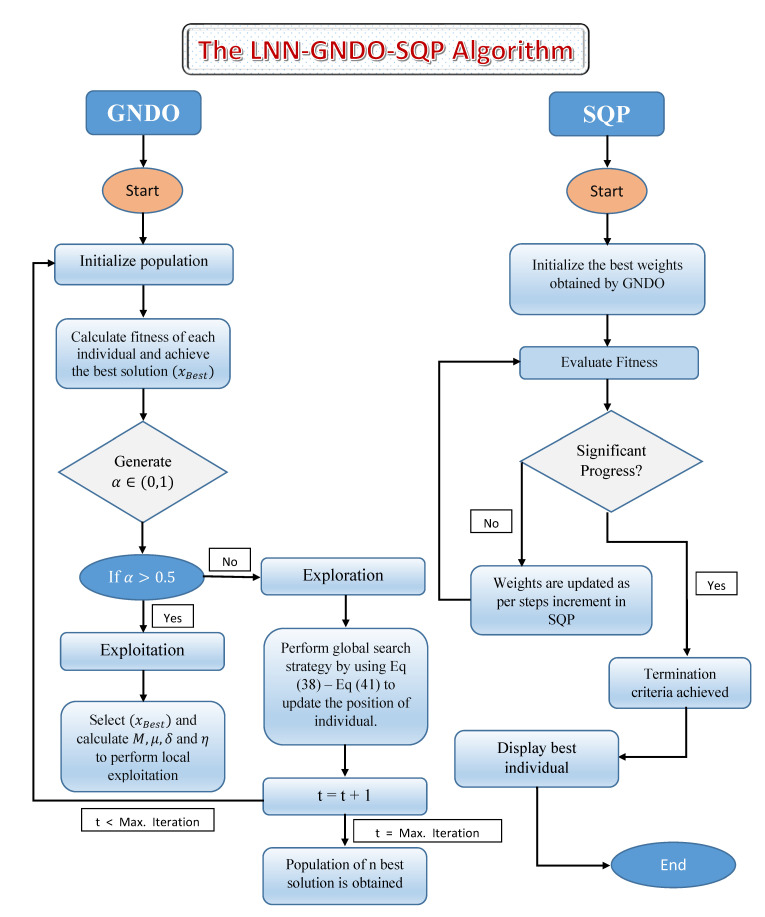
Flowchart of the LNN-GNDO-SQP Algorithm.

A novel methodology is adopted for calculating unknown weights in LNN by optimizing a fitness function; see Equation (35) for the drainage problem using a hybrid procedure of a generalized normal distribution optimization (GNDO) technique and sequential quadratic programming (SQP). An illustrative flowchart of the LNN-GNDO-SQP algorithm is given in Figure 3.

#### 3.3.1. Brief Introduction of Generalized Normal Distribution Optimization (GNDO) Algorithm

The generalized normal distribution optimization technique was developed by Zhang et al. [36], a novel metaheuristic algorithm inspired by normal distribution theory in which each individual’s location is modified using a generalized normal curve for distribution. The GNDO algorithm is an effective and efficient technique for finding optimal solutions for constrained and unconstrained optimization problems by enhancing and extracting the precision of unknown parameters. The working framework of the GNDO algorithm is classified into two main categories, named exploitation and exploration. The two phases are of equal importance in GNDO, and have the same probability of being chosen in order to optimize a problem.

(a) Exploitation: It is a process of finding optimum solutions around the candidate space containing the current position of individuals. This phase’s working procedure is based on a relation between the normal distribution of population and the distribution of each individual in a population. The model for optimization can be expressed as:

(37) $$u_{i}^{t}=\delta_{i}\times \eta +\mu_{i},\;i=1,2,3,\ldots ,N,$$

where $\delta_{i},\eta$ and $\mu_{i}$ are defined as:

(38) $$\delta_{i}=\sqrt{\frac{1}{3}\left[{\left(x_{i}^{t}-\mu \right)}^{2}+{\left(M-\mu \right)}^{2}+{\left(x_{Best}^{t}-\mu \right)}^{2}\right]},$$

(39) $$\eta =\left\{\begin{array}{c} \sqrt{-\log\left(\lambda_{1}\right)}\times \cos\left(2\pi \lambda_{2}\right),\;\mathrm{if}\;a\leq b,\\ \sqrt{-\log\left(\lambda_{1}\right)}\times \cos\left(2\pi \lambda_{2}+\pi \right),\;\mathrm{otherwise},\; \end{array}\right.$$

(40) $$\mu_{i}=\frac{1}{3}\left(x_{i}^{t}+x_{Best}^{t}+M\right),$$

$u^{t}$, $\mu_{i}$ and $\delta_{i}$ are trial vector, generalized mean and generalized variance of ith individual at any time *t*. Penalty factor is denoted by η. $a,b,\lambda_{1}$ and $\lambda_{2}$ are randomly chosen numbers between 0 and 1. $x_{Best}^{t}$, which is the so far best position of individual while its mean position is denoted by *M* and defined as:

(41) $$M=\frac{\sum_{i=1}^{N}x_{i}^{t}}{N}.$$

(b) Exploration: It is a process of searching a population space globally to find promising solutions. The global search phase in GNDO is subjected to three arbitrarily selected individuals, which are modeled as:

(42) $$u_{i}^{t}=x_{i}^{t}+\beta \times \left(\left|\lambda_{3}\right|\times u_{1}\right)+\left(1-\beta \right)\times \left(\left|\lambda_{4}\right|\times u_{2}\right),$$

local information is presented by $x_{i}^{t}+\beta \times \left(\left|\lambda_{3}\right|\times v_{1}\right)$ while global information is shared by $\left(1-\beta \right)\times \left(\left|\lambda_{4}\right|\times v_{2}\right)$. $u_{1}$ and $u_{2}$ are trail vectors while β is adjustment parameter between 0 and 1. $u_{1}$ and $u_{2}$ can be calculated as

(43) $$u_{1}=\left\{\begin{array}{c} x_{i}^{t}-x_{\mathrm{pl}}^{t},\;\mathrm{if}\;f\left(x_{i}^{t}\right)<f\left(x_{p1}^{t}\right),\\ x_{p1}^{t}-x_{i}^{t},\;\mathrm{otherwise}\;, \end{array}\right.$$

and

(44) $$u_{2}=\left\{\begin{array}{c} x_{p2}^{t}-x_{p3}^{t},\;\mathrm{if}\;f\left(x_{p2}^{t}\right)<f\left(x_{p3}^{t}\right),\\ x_{p3}^{t}-x_{p2}^{t},\;\mathrm{otherwise}\;, \end{array}\right.$$

where p1≠p2≠p3≠i are integers between 1 to N.

#### 3.3.2. Sequential Quadratic Programming

To enhance the local search characteristic of our solution technique, we have combined SQP with GNDO. SQP is a well-established single path following the local search algorithm, which has considerably enhanced our algorithm’s convergence speed. SQP is considered a classical method developed in 1963 for solving constrained/unconstrained linear and nonlinear optimization problems [37]. The SQP algorithm is known to be one of the basic approaches to be used in the context of public and commercial sector problems of significant importance. Some recent applications of SQP include numerical simulation of shakedown analysis of structure [38], economic load dispatch problem [39] and turbulent flow [40].

### 3.4. Hybridization of GNDO-SQP Algorithm

Working procedure of LNN-GNDO-SQP algorithm is summarized as:

**Step 1**Initialization: Trial solution Equation (30) for drainage problem is considered and unknown parameters of LNN are initialized with randomly generated real values for population space. Mathematically, individuals can be listed as:

(45) $$C=\left[\left(\alpha ,\omega ,\beta \right)\right]=\left[\alpha_{1},\alpha_{2},\ldots \alpha_{n},\omega_{1},\omega_{2},\ldots \omega_{n},\beta_{1},\beta_{2},\ldots \beta_{n}\right],$$

where *n* denotes the number of weights in LNN model. Parameter setting for GNDO algorithm is given in Table 1.

**Step 2**Fitness calculation: GNDO evaluates the fitness function Equation (35) and updates the weights when required until termination criteria is achieved.

**Step 3**Ranking: Unknown parameters attained with GNDO for error based function (fitness function) during multiple runs are ranked in ascending order.

**Step 4**Initializing SQP: The unknown parameters corresponding to minimum value of fitness function achieved by GNDO are considered as the initial guess or initial weights to supervise sequential quadratic programming.

**Step 5**Fitness calculation: Fitness function for drainage problem is calculated by using SQP algorithm with updated weights of GNDO.

**Step 6**Stopping criteria: The weights are updated with GNDO-SQP algorithm and the process is stopped when minimum criteria on fitness value is achieved. Parameter settings for SQP is given in Table 1.

**Step 7**Storage: Weights, fitness value, residual and absolute errors are stored for the optimization of drainage problem. Graphical overview of the LNN-GNDO-SQP algorithm given in Figure 3.

The LNN-GNDO-SQP algorithm has a simple structure and easy to implement. The GNDO algorithm updates the position of individual using generalized normal distribution formula and SQP complements its local convergence. Since Legendre polynomials are orthogonal on [−1,1], the experimental analysis shows that proposed algorithm converges to best solutions for number of real world problems by training the weights from the interval [−1,1]. It has been noticed that the convergence of the design scheme is slightly affected by increasing the domain.

Computational complexity analysis (CCA) is evaluated for the proposed algorithm based on the average time taken to calculate unknown neurons in the LNN structure using the GNDO-SQP algorithm. Values of complexity operators for different drainage problem scenarios show execution time and fitness evaluation in terms of mean and standard deviations are dictated in Table 2. The results show the consistency of the proposed algorithm. All calculations and evaluations for this research were conducted on an HP laptop Elitebook 840 G2 with intel(R) Core(TM) i5-5300 CPU @ 2.30 GHz, 8.00 GB RAM, 64 bit operating in Microsoft Windows 10 Education edition running the R2018a version of MATLAB.

## 4. Experimental Setup and Statistical Evaluation

In this section, the statistical analysis of different drainage problems based on mean, standard deviation and minimum values is presented. Performance indications like Mean absolute deviation (MAD), Theil’s inequality coefficient (TIC), Root mean square error (RMSE), and Error in Nash Sutcliffe efficiency (ENSE) are defined to study the efficiency of the proposed algorithm. Global performance indicators like GMAD, GTIC, GRMSE, and GENSE are also defined to study our novel computing approach’s overall performance. Formulation of performance metrics are given as:

(46) $$MAD=\frac{1}{n}\sum_{i=1}^{n}\left(\left|w\left(r_{i}\right)-w_{approx}\left(r_{i}\right)\right|\right),$$

(47) $$TIC=\frac{\sqrt{\frac{1}{n}\sum_{i=1}^{n}{\left(w\left(r_{i}\right)-w_{approx}\left(r_{i}\right)\right)}^{2}}}{\sqrt{\frac{1}{n}\sum_{i=1}^{n}{\left(w\left(r_{i}\right)\right)}^{2}}+\sqrt{\frac{1}{n}\sum_{i=1}^{n}{\left(w_{approx}\left(r_{i}\right)\right)}^{2}}},$$

(48) $$RMSE=\frac{1}{n}\sqrt{\sum_{i=1}^{n}{\left(w\left(r_{i}\right)-w_{approx}\left(r_{i}\right)\right)}^{2}},$$

(49) $$\mathrm{NSE}=\left\{1-\frac{\sum_{i=1}^{n}{\left(w\left(r_{i}\right)-w_{approx}\left(r_{i}\right)\right)}^{2}}{\sum_{i=1}^{n}{\left(w\left(r_{i}\right)-\bar{w}\left(r_{i}\right)\right)}^{2}}\right.,\;\bar{w}\left(r_{i}\right)=\sum_{i=1}^{n}\left(w\left(r_{i}\right)\right),$$

(50) ENSE=|1−NSE|,

here, $\hat{w}\left(r_{i}\right)=w_{approx}\left(r_{i}\right)$.

In the case of a perfect solution, the values of these performance indices must approach zero. The formulations of global performance indices are given as:

(51) $$GMAD=\frac{1}{R_{n}}\sum_{j=1}^{R_{n}}MAD,\;GTIC=\frac{1}{R_{n}}\sum_{j=1}^{R_{n}}TIC,\;GRMSE=\frac{1}{R_{n}}\sum_{j=1}^{R_{n}}RMSE,\;GENSE=\frac{1}{R_{n}}\sum_{j=1}^{R_{n}}ENSE,$$

where $R_{n}$ denotes the number of independent runs.

### Numerical Simulation and Discussion

**Figure 4:**
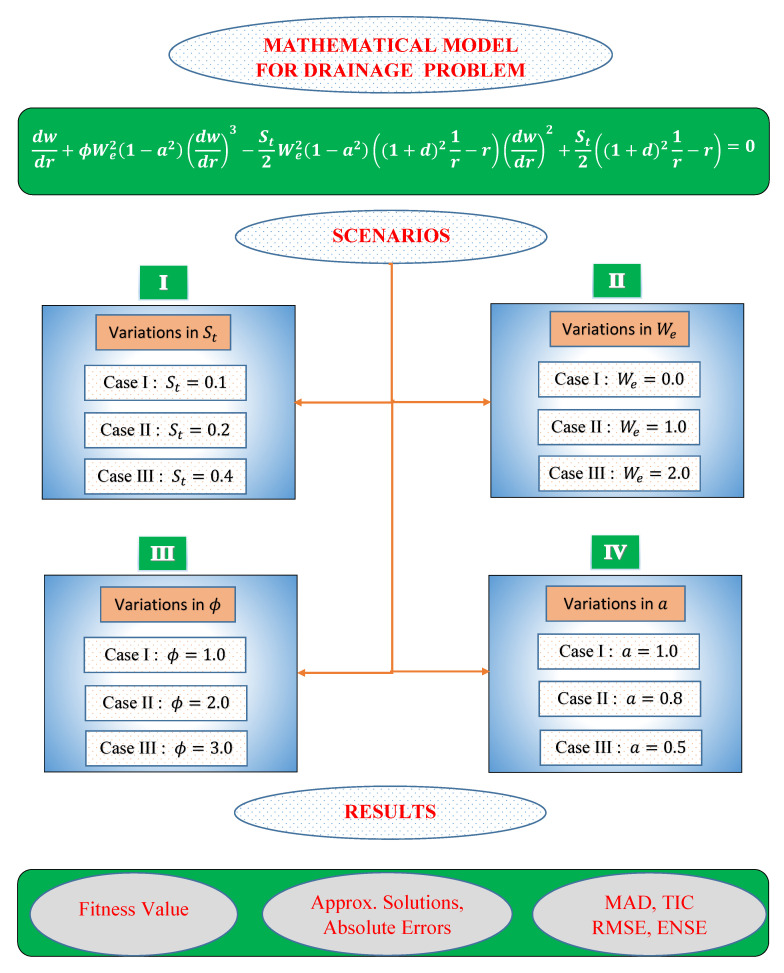
Graphical overview of the problem considered in this paper.

In this section, different scenarios of non-linear drainage problem is considered with different cases depending on the variations in parameters like $S_{t}$, $W_{e}$, ϕ and *a*. The detailed overview of the problems discussed in the paper are shown through Figure 4.

SCENARIO I: Effect of variation in Stokes number $S_{t}$ on drainage problem. Following three cases are considered **CASE I**: $S_{t}=0.1$, CASE II: $S_{t}=0.3$ and CASE III: $S_{t}=0.4$ where $W_{e}=\phi =1$, a=0.1 and thickness δ=1.

Fitness function of Equation (35) for the scenario I is formulated as

(52) $$\varepsilon =\frac{1}{n}\sum_{m=1}^{n}{\left(\begin{array}{c} \frac{d{\hat{w}}_{m}}{dr}+0.99{\left(\frac{d{\hat{w}}_{m}}{dr}\right)}^{3}-0.495S_{t}\left(\frac{4}{r}-r\right){\left(\frac{d{\hat{w}}_{m}}{dr}\right)}^{2}-\frac{S_{t}}{2}\left(\frac{4}{r}-r\right) \end{array}\right)}^{2}+{\left(w(1)\right)}^{2}.$$

SCENARIO II: Effect of variation in Weissenberg number $W_{e}$ on drainage problem. Following three cases are considered CASE I: $W_{e}=0.0$, CASE II: $W_{e}=1.0$ and CASE III: $W_{e}=2.0$ where $S_{t}=0.5$, ϕ=0.80, a=0.50 and thickness δ=1.0.

Fitness function of Equation (35) for the scenario II is formulated as

(53) $$\varepsilon =\frac{1}{n}\sum_{m=1}^{n}{\left(\begin{array}{c} \frac{d{\hat{w}}_{m}}{dr}+0.6W_{e}^{2}{\left(\frac{d{\hat{w}}_{m}}{dr}\right)}^{3}-0.1875W_{e}^{2}\left(\frac{4}{r}-r\right){\left(\frac{d{\hat{w}}_{m}}{dr}\right)}^{2}-0.25\left(\frac{4}{r}-r\right) \end{array}\right)}^{2}+{\left(w(1)\right)}^{2}.$$

SCENARIO III: Effect of variation in ratio of viscosities ϕ on drainage problem. Following three cases are considered CASE I: ϕ=1.0, CASE II: ϕ=2.0 and CASE III: ϕ=3.0 where $S_{t}=0.5$, $W_{e}=1.0$, a=0.50 and thickness δ=1.0.

Fitness function of Equation (35) for the scenario III is formulated as

(54) $$\varepsilon =\frac{1}{n}\sum_{m=1}^{N}{\left(\begin{array}{c} \frac{d{\hat{w}}_{m}}{dr}+0.75\phi {\left(\frac{d{\hat{w}}_{m}}{dr}\right)}^{3}-0.1875\left(\frac{4}{r}-r\right){\left(\frac{d{\hat{w}}_{m}}{dr}\right)}^{2}-0.25\left(\frac{4}{r}-r\right) \end{array}\right)}^{2}+{\left(w(1)\right)}^{2}.$$

SCENARIO IV: Effect of variation in slip parameter *a* on drainage problem. Following three cases are considered CASE I: a=1.0, **CASE II**: a=0.8 and CASE III: a=0.5 where $S_{t}=0.70$, $W_{e}=1.30$, ϕ=0.60 and thickness δ=1.0.

Fitness function of Equation (35) for the scenario IV is formulated as

(55) $$\varepsilon_{1}=\frac{1}{n}\sum_{m=1}^{n}{\left(\begin{array}{c} \frac{d{\hat{w}}_{m}}{dr}+1.014\left(1-a^{2}\right){\left(\frac{d{\hat{w}}_{m}}{dr}\right)}^{3}-0.5915\left(1-a^{2}\right)\left(\frac{4}{r}-r\right){\left(\frac{d{\hat{w}}_{m}}{dr}\right)}^{2}-0.35\left(\frac{4}{r}-r\right) \end{array}\right)}^{2}+{\left(w(1)\right)}^{2}.$$

In this paper, the mathematical model of the thin film flow of non Newtonian fluid is formulated for drainage. Four scenarios are considered depending on the variations in parameters, including Stokes number $S_{t}$, Weissenberg number $W_{e}$, a ratio of viscosities ϕ, and slip parameter *a*. A novel soft computing technique is developed to solve the non-linear mathematical model for drainage problem, see Equation (29). Approximate solutions obtained by our proposed technique, the LNN-GNDO-SQP algorithm, are compared with MATLAB solver RK-4 and hybrid of genetic algorithm and active set algorithm (GA-ASA) [27].

**Figure 5:**
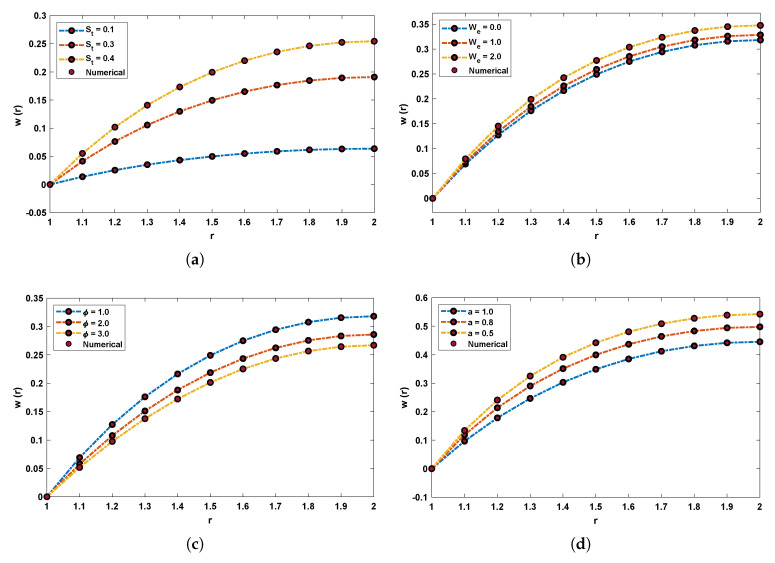
Comparison of dimensionless velocity profile obtained by LNN-GNDO-SQP algorithm with RK-4 method for variants of drainage problem. (**a**) SCENARIO I. (**b**) SCENARIO II. (**c**) SCENARIO III. (**d**) SCENARIO IV.

Fitness functions as in Equations (52)–(55) corresponding to four scenarios of the drainage problem are optimized by performing 100 independent runs using the LNN-GNDO-SQP algorithm. It is evident from Figure 5 that best approximated solutions obtained by proposed scheme overlaps the numerical and GA-ASA solutions for each scenario. Furthermore, it can be seen from Figure 5a,b that with increase in Stokes and Weissenberg number the velocity profile w(r) of Johnson–Segalman fluid increases while from Figure 5c,d it is observed that w(r) decreases with an increase in the ratio of viscosity and slip parameters. The magnitude of the velocity of the fluid decreases as the fluid becomes thicker and vice versa. Also, the Newtonian flow behaviour of the fluid was observed for $W_{e}=0.0$, α=1 and ϕ=1.0.

**Figure 6:**
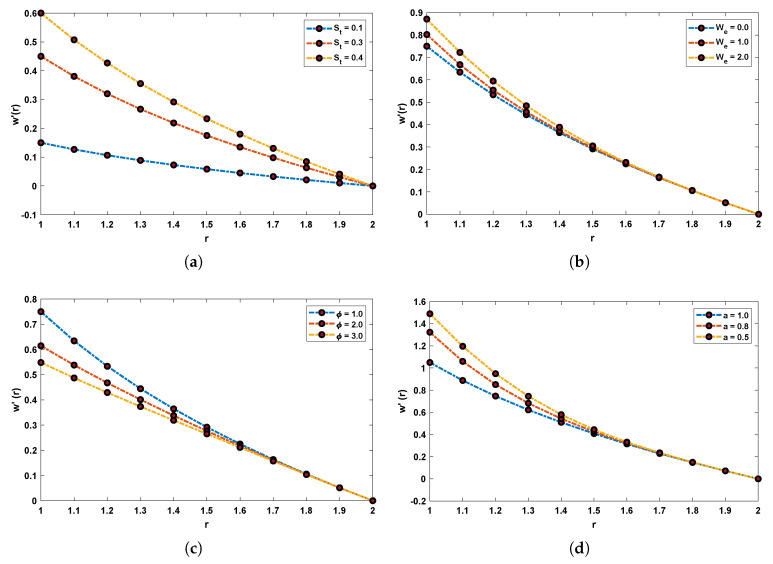
Effect of variations in $S_{t}$, $W_{e}$, ϕ and *a* on $w^{\prime }$ of the flow of Johnson–Segalman fluid. (**a**) SCENARIO I. (**b**) SCENARIO II. (**c**) SCENARIO III. (**d**) SCENARIO IV.

**Figure 7:**
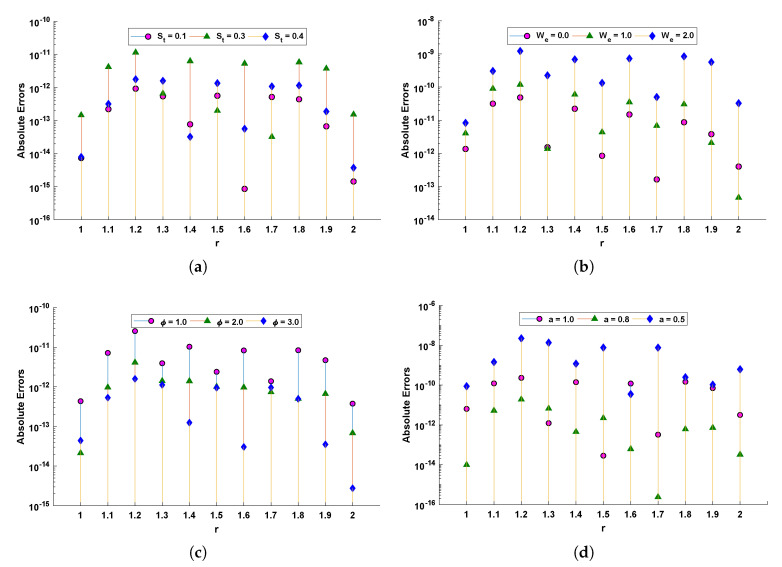
Absolute error in solutions calculated by the LNN-GNDO-SQP algorithm for different scenarios of drainage problem. (**a**) SCENARIO I. (**b**) SCENARIO II. (**c**) SCENARIO III. (**d**) SCENARIO IV.

**Figure 8:**
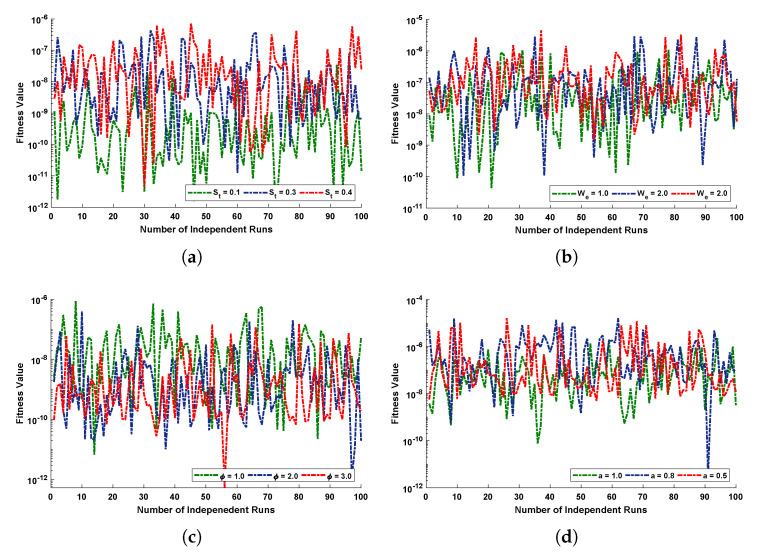
Behaviour of fitness value during the 100 executions of the proposed LNN-GNDO-SQP algorithm for various scenarios of the flow of Johnson Segalman fluid on the surface of an infinitely long vertical cylinder. (**a**) SCENARIO I. (**b**) SCENARIO II. (**c**) SCENARIO III. (**d**) SCENARIO IV.

Graphical illustration for performance of the designed algorithm in obtaining change in velocity profile and absolute errors in best solutions for all scenarios are shown in Figure 6 and Figure 7. The convergence of fitness values for each scenario with different cases is shown in Figure 8. Approximate solution and absolute errors in the best solution for each scenario are listed in Table 3, Table 4, Table 5 and Table 6. Percentage errors are calculated for each case of scenario I, II, III and IV. The average %Errors for different cases of each scenario are 0.36%, 0.58%, 0.53%, 0.74%, 0.86%, 0.60%, 0.57%, 0.20%, 0.17%, 1.01%, 0.63% and 1.00% respectively. It is obvious from these errors that our approach is successful in calculating best solutions with less errors. Weights obtained by proposed scheme for optimization of fitness functions (Equations (52)–(55)) are given in Table 7, Table 8, Table 9 and Table 10. These weights are useful in producing our results.

**Figure 9:**
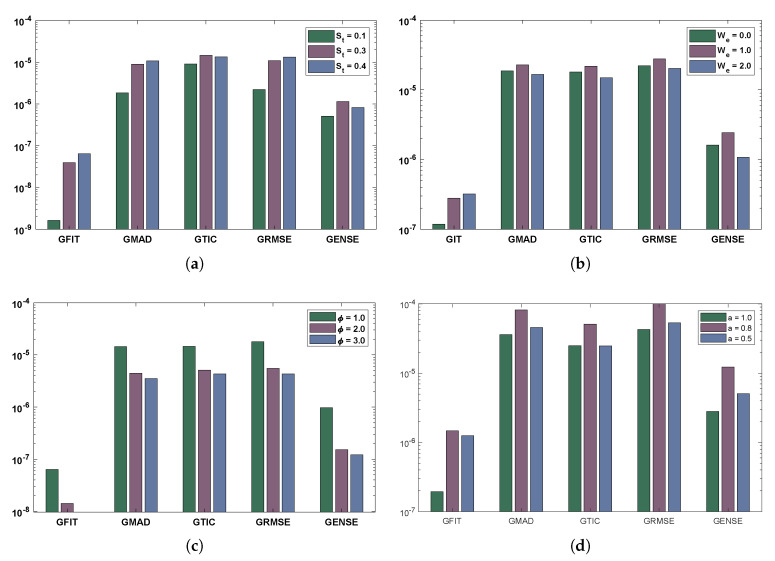
Global performance indicators for four scenarios of the drainage problem. (**a**) SCENARIO I. (**b**) SCENARIO II. (**c**) SCENARIO III. (**d**) SCENARIO IV.

**Figure 10:**
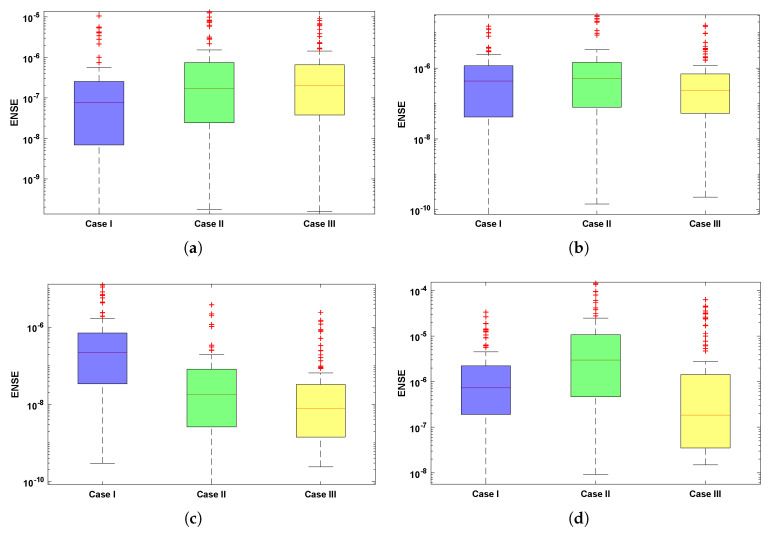
Boxplots showing distribution of ENSE values for four scenarios of the drainage problem. (**a**) SCENARIO I. (**b**) SCENARIO II. (**c**) SCENARIO III. (**d**) SCENARIO IV.

The comparison of statistical data for absolute errors in terms of minimum, mean, and standard deviation for scenario I obtained by the LNN-GNDO-SQP algorithm are compared with GA-ASA [27] as shown in Table 11. It can be seen that minimum values, mean and standard deviation at each step size r=0.05 obtained by the design algorithm dominates the results available in the latest literature [27]. Statistical data of MAD, TIC, RMSE, and ENSE in terms of minimum, mean and standard deviation are presented in Table 12, Table 13, Table 14 and Table 15. Minimum values of fitness, MAD, TIC, RMSE and ENSE for different cases of each scenarios lies around $10^{-11}$ to $10^{-12}$, $10^{-6}$ to $10^{-7}$, $10^{-6}$ to $10^{-7}$ and $10^{-9}$ to $10^{-11}$ respectively. Bar and box graphs are plotted through Figure 9 and Figure 10 to illustrate values of the global performance indicators such as MAD, TIC, RMSE, and ENSE. Global values of performance indices lie between $10^{-6}$ and $10^{-7}$, which establishes our algorithm’s superiority. The series solutions for each of scenario I, II, III and IV are given in Appendix A.

## 5. Conclusions

This paper has investigated the steady thin film flow of non-Newtonian fluid on the vertical cylinder’s outer surface used in drainage problems. The drainage problem is mathematically modeled using basic concepts of continuity and a momentum equation that results in partial differential equations. Furthermore, the problem is reduced to a nonlinear ordinary differential equation by incorporating a similarity transformation technique. To study the velocity profile of Johnson–Segalman fluid under the influence of variations in Stokes number, Weissenberg numbers, ratio of viscosities, and slip parameter, a novel soft computing technique is designed in which Legendre polynomials are weighted with neurons in artificial neural networks (ANN) that is used to model approximate solutions for Equation (29).

In order to determine the accuracy of candidate solutions, fitness functions are constructed. We have used an efficient global search mechanism, namely, generalized normal distribution optimization (GNDO), and a local search technique known as sequential quadratic programming algorithm to minimize the fitness function. Better approximate series solutions are calculated for different cases of each scenario shown in Figure 5. Our results are validated by comparing the statistical values of absolute errors in terms of minimum, mean, and standard deviation obtained by the proposed algorithm with the GA-ASA algorithm as shown in Table 11.

We have investigated the variations in velocity profile due to variations in Stokes number, Weissenberg numbers, ratio of viscosities and slip parameter. It is established that changes in velocity profile are directly proportional to Stokes number and Weissenberg numbers. In contrast, variations in the ratio of viscosities and slip parameters have an inverse relation with the velocity profile. Extensive statistical and graphical analysis for drainage problems illustrates the stability, efficiency, and effectiveness of the proposed algorithm in solving real-world problems.

In the future, the LNN-GNDO-SQP algorithm can be used to solve problems involving systems of differential equations. Furthermore, the given scheme’s applicability domain can be readily extended to solve tumor growth and HIV infection dynamics, financial mathematics problems, econometric problems, optimal control models, and many other systems where convention methodologies fail.

## Figures and Tables

**Table 1:** Parameter settings for GNDO and SQP algorithms.

**Parameter**	**Setting**	**Parameter**	**Setting**
Algorithm	GNDO	Bounds [lower, upper]	[−1,1]
Maximum Iterations	6000	X-tolerance (TolX)	$10^{-20}$
Maximum function evaluations	150,000	Search Agents	70
Fitness	$10^{-20}$	Function tolerance (TolFun)	$10^{-20}$
Algorithm	SQP	Bounds [lower, upper]	[−1,1]
Maximum Iterations	3000	Function tolerance (TolFun)	$10^{-18}$
Maximum function evaluations	200,000	X-tolerance (TolX)	$10^{-20}$

**Table 2:** Computational complexity analysis of the proposed algorithm for different cases of the drainage problem.

		**Time (s)**				**Fitness Evaluation**			
		**GNDO**		**SQP**		**LNN-GNDO-SQP**		**LNN-GNDO-SQP**	
**Scenarios**	**Cases**	**Mean**	**Std**	**Mean**	**Std**	**Mean**	**Std**	**Mean**	**Std**
	I	18.4108	3.4887	4.5928	0.0705	23.8861	3.8476	89,004.8	16,731.3
I	II	19.0551	3.3897	4.5532	0.0368	24.8434	3.3793	93,340.1	14,831.1
	III	20.2797	4.5199	8.6643	0.8056	30.4264	5.2021	92,509.5	9581.3
	I	22.2363	3.3865	5.5877	0.099	29.3197	3.3619	90,013.6	12,904.2
II	II	21.7863	2.9221	5.5285	0.1559	28.8605	2.8051	90,002.5	12,910.1
	III	22.8682	6.1756	5.3493	0.1791	28.0621	3.7356	96,845.3	6833.2
	I	19.2665	2.1826	4.7025	0.5069	25.2665	1.9573	92,503.7	17,079.5
III	II	22.0751	3.4185	5.5411	0.1166	29.1795	3.433	90,014.1	11,906.1
	III	21.8293	4.0707	5.3898	0.2011	28.8158	4.2539	93,378.8	9146.3
	I	20.5727	3.4724	5.4403	0.1086	27.6455	3.6496	97,547.2	12,852.6
IV	II	22.3783	4.5886	5.5063	0.1848	29.5412	4.7723	91,364.9	8806.3
	III	20.1867	1.9982	5.6793	0.2979	27.4167	2.2246	95,010.9	8228.1

**Table 3:** Approximate solutions and Absolute errors obtained by the LNN-GNDO-SQP algorithm for SCENARIO I of the drainage problem.

	Solutions			Absolute Errors		
*r*	Case I	Case II	Case III	Case I	Case II	Case III
1.0	$-1.17\times 1^{-09}$	$-2.93\times 10^{-08}$	$6.62\times 10^{-10}$	$7.36\times 10^{-15}$	$1.46\times 10^{-13}$	$7.92\times 10^{-15}$
1.1	0.013812	0.041437	0.055248	$2.22\times 10^{-13}$	$4.19\times 10^{-12}$	$3.18\times 10^{-13}$
1.2	0.025464	0.076393	0.101857	$9.24\times 10^{-13}$	$1.15\times 10^{-11}$	$1.77\times 10^{-12}$
1.3	0.035223	0.105669	0.140892	$5.42\times 10^{-13}$	$6.59\times 10^{-13}$	$1.59\times 10^{-12}$
1.4	0.043295	0.129884	0.173178	$7.71\times 10^{-14}$	$6.31\times 10^{-12}$	$3.22\times 10^{-14}$
1.5	0.049843	0.149529	0.199372	$5.64\times 10^{-13}$	$1.99\times 10^{-13}$	$1.35\times 10^{-12}$
1.6	0.055001	0.165002	0.220003	$8.59\times 10^{-16}$	$5.31\times 10^{-12}$	$5.64\times 10^{-14}$
1.7	0.058876	0.176627	0.235503	$5.17\times 10^{-13}$	$3.23\times 10^{-14}$	$1.08\times 10^{-12}$
1.8	0.061557	0.184672	0.246230	$4.41\times 10^{-13}$	$5.84\times 10^{-12}$	$1.15\times 10^{-12}$
1.9	0.063121	0.189363	0.252483	$6.69\times 10^{-14}$	$3.74\times 10^{-12}$	$1.88\times 10^{-13}$
2.0	0.063630	0.190888	0.254518	$1.44\times 10^{-15}$	$1.53\times 10^{-13}$	$3.73\times 10^{-15}$

**Table 4:** Approximate solutions and Absolute errors obtained by the LNN-GNDO-SQP algorithm for SCENARIO II of the drainage problem.

	Solutions			Absolute Errors		
*r*	Case I	Case II	Case III	Case I	Case II	Case III
1.0	$1.60\times 10^{-08}$	$3.22\times 10^{-07}$	$-3.79\times 10^{-08}$	$1.36\times 10^{-12}$	$4.02\times 10^{-12}$	$8.29\times 10^{-12}$
1.1	0.069061	0.073286	0.079469	$3.16\times 10^{-11}$	$9.00\times 10^{-11}$	$3.04\times 10^{-10}$
1.2	0.127322	0.134214	0.145147	$4.85\times 10^{-11}$	$1.19\times 10^{-10}$	$1.22\times 10^{-09}$
1.3	0.176115	0.184630	0.198951	$1.53\times 10^{-12}$	$1.37\times 10^{-12}$	$2.25\times 10^{-10}$
1.4	0.216473	0.225933	0.242478	$2.22\times 10^{-11}$	$5.98\times 10^{-11}$	$6.80\times 10^{-10}$
1.5	0.249216	0.259191	0.277061	$8.46\times 10^{-13}$	$4.37\times 10^{-12}$	$1.33\times 10^{-10}$
1.6	0.275004	0.285236	0.303803	$1.49\times 10^{-11}$	$3.50\times 10^{-11}$	$7.24\times 10^{-10}$
1.7	0.294379	0.304720	0.323602	$1.64\times 10^{-13}$	$6.82\times 10^{-12}$	$4.98\times 10^{-11}$
1.8	0.307788	0.318167	0.337161	$8.70\times 10^{-12}$	$3.03\times 10^{-11}$	$8.39\times 10^{-10}$
1.9	0.315605	0.325992	0.345010	$3.80\times 10^{-12}$	$2.07\times 10^{-12}$	$5.67\times 10^{-10}$
2.0	0.318148	0.328535	0.347552	$4.01\times 10^{-13}$	$4.65\times 10^{-14}$	$3.25\times 10^{-11}$

**Table 5:** Approximate solutions and Absolute errors obtained by the LNN-GNDO-SQP algorithm for SCENARIO III of the drainage problem.

	Solutions			Absolute Errors		
*r*	Case I	Case II	Case III	Case I	Case II	Case III
1.0	$2.78\times 10^{-08}$	$1.20\times 10^{-09}$	$2.25\times 10^{-08}$	$4.35\times 10^{-13}$	$2.14\times 10^{-14}$	$4.41\times 10^{-14}$
1.1	0.069061	0.057570	0.051729	$7.19\times 10^{-12}$	$9.73\times 10^{-13}$	$5.33\times 10^{-13}$
1.2	0.127322	0.107809	0.097514	$2.55\times 10^{-11}$	$4.13\times 10^{-12}$	$1.59\times 10^{-12}$
1.3	0.176115	0.151207	0.137633	$3.95\times 10^{-12}$	$1.41\times 10^{-12}$	$1.12\times 10^{-12}$
1.4	0.216473	0.188122	0.172252	$1.03\times 10^{-11}$	$1.40\times 10^{-12}$	$1.25\times 10^{-13}$
1.5	0.249216	0.218821	0.201457	$2.40\times 10^{-12}$	$1.01\times 10^{-12}$	$9.50\times 10^{-13}$
1.6	0.275004	0.243515	0.225278	$8.30\times 10^{-12}$	$9.73\times 10^{-13}$	$3.04\times 10^{-14}$
1.7	0.294379	0.262388	0.243717	$1.39\times 10^{-12}$	$7.36\times 10^{-13}$	$9.64\times 10^{-13}$
1.8	0.307787	0.275618	0.256782	$8.39\times 10^{-12}$	$5.05\times 10^{-13}$	$4.97\times 10^{-13}$
1.9	0.315604	0.283396	0.264522	$4.71\times 10^{-12}$	$6.68\times 10^{-13}$	$3.53\times 10^{-14}$
2.0	0.318148	0.285937	0.267060	$3.78\times 10^{-13}$	$6.83\times 10^{-14}$	$2.76\times 10^{-15}$

**Table 6:** Approximate solutions and Absolute errors obtained by the LNN-GNDO-SQP algorithm for SCENARIO IV of the drainage problem.

	Solutions			Absolute Errors		
*r*	Case I	Case II	Case III	Case I	Case II	Case III
1.0	$3.30\times 10^{-08}$	$1.39\times 10^{-07}$	$8.89\times 10^{-06}$	$6.40\times 10^{-12}$	$9.89\times 10^{-15}$	$8.73\times 10^{-11}$
1.1	0.096686	0.118626	0.133905	$1.22\times 10^{-10}$	$5.24\times 10^{-12}$	$1.43\times 10^{-09}$
1.2	0.178252	0.213761	0.240778	$2.32\times 10^{-10}$	$1.91\times 10^{-11}$	$2.21\times 10^{-08}$
1.3	0.246561	0.290113	0.325136	$1.23\times 10^{-12}$	$6.64\times 10^{-12}$	$1.35\times 10^{-08}$
1.4	0.303063	0.351164	0.391060	$1.40\times 10^{-10}$	$4.51\times 10^{-13}$	$1.18\times 10^{-09}$
1.5	0.348903	0.399427	0.441979	$2.86\times 10^{-14}$	$2.21\times 10^{-12}$	$7.60\times 10^{-09}$
1.6	0.385007	0.436712	0.480563	$1.20\times 10^{-10}$	$6.22\times 10^{-14}$	$3.52\times 10^{-11}$
1.7	0.412130	0.464339	0.508748	$3.26\times 10^{-13}$	$2.41\times 10^{-16}$	$7.55\times 10^{-09}$
1.8	0.430903	0.483280	0.527879	$1.47\times 10^{-10}$	$6.13\times 10^{-13}$	$2.46\times 10^{-10}$
1.9	0.441847	0.494259	0.538894	$7.02\times 10^{-11}$	$7.23\times 10^{-13}$	$1.05\times 10^{-10}$
2.0	0.445406	0.497823	0.542465	$3.18\times 10^{-12}$	$3.27\times 10^{-14}$	$6.17\times 10^{-10}$

**Table 7:** Best set of weights obtained by the LNN-GNDO-SQP algorithm using a fitness function as in Equation (52). Here three cases are studied by varying $S_{t}$ in the drainage problem.

	Case I			Case II			Case III		
Index	$\alpha_{j}$	$\omega_{j}$	$\beta_{j}$	$\alpha_{j}$	$\omega_{j}$	$\beta_{j}$	$\alpha_{j}$	$\omega_{j}$	$\beta_{j}$
1	−0.267240	−0.002610	0.304056	−0.161130	0.245666	0.153137	−0.642690	−0.817430	0.856846
2	0.199856	0.083281	0.361633	0.540048	−0.082950	−0.133580	−0.500960	−0.085350	0.282074
3	0.574441	−0.211200	−0.000280	0.999999	0.004495	0.031383	−0.502540	−0.411400	0.346607
4	0.939992	0.009259	−0.304090	−0.609000	−0.206300	−0.187450	0.986752	−0.145940	0.599804
5	−0.411700	0.102550	0.009940	−0.952670	−0.488120	0.153461	0.627584	0.615380	−0.986650
6	−0.280860	0.091810	−0.271960	0.042458	0.321851	−0.117770	0.271924	−0.325700	−0.085320
7	0.172679	−0.600230	0.705329	−0.101030	−0.283540	0.999321	0.243758	0.265845	−0.090110
8	0.065151	0.313916	0.079046	−0.277050	0.204757	0.353785	−0.863580	−0.368860	0.070886
9	0.159743	−0.031440	0.179555	0.373401	−0.330670	0.288077	−0.155260	−0.116030	−0.412520
10	0.433358	−0.300480	0.174122	0.363993	0.240191	0.020892	0.825776	−0.133910	0.119581
11	−0.362530	−0.225430	0.133951	0.999719	0.041990	0.288770	−0.148830	−0.229960	−0.050250

**Table 8:** Best set of weights obtained by the LNN-GNDO-SQP algorithm using a fitness function as in Equation (53). Here three cases are studied by varying $W_{e}$ in the drainage problem.

	Case I			Case II			Case III		
Index	$\alpha_{j}$	$\omega_{j}$	$\beta_{j}$	$\alpha_{j}$	$\omega_{j}$	$\beta_{j}$	$\alpha_{j}$	$\omega_{j}$	$\beta_{j}$
1	−0.277180	−0.928610	0.990229	−0.984680	−0.867220	0.370173	−0.575380	−0.988420	0.997400
2	−0.744240	0.990077	−0.999960	−0.801200	0.555550	−0.024790	−0.999990	0.131423	0.343622
3	−0.553260	−0.988500	0.923460	−0.002580	0.129594	−0.998370	0.143249	−0.854240	0.140163
4	−0.404730	0.398152	0.127399	−0.047010	0.700149	−0.999990	−0.119360	0.373826	0.207802
5	−0.809510	0.036150	0.140306	−0.690450	0.398839	−0.196860	−0.856660	−0.352810	0.010636
6	0.698433	0.464628	0.151832	0.301364	0.237875	0.160810	−0.797780	−0.322780	0.999999
7	−0.003180	0.192692	0.395066	0.423747	0.272096	−0.176540	−0.645030	−0.182700	−0.655790
8	−0.150320	−0.098370	−0.558090	−0.997780	−0.212630	−0.417770	0.012084	0.257723	−0.149790
9	−0.992840	−0.329570	0.734408	0.274115	−0.255500	0.001304	−0.753090	−0.306120	0.466878
10	0.182691	−0.066350	−0.374700	−0.428760	−0.199540	0.929899	0.498251	0.125154	0.236910
11	−0.629380	−0.361980	0.101130	−0.138360	−0.138270	0.103016	0.079987	0.174965	0.126845

**Table 9:** Best set of weights obtained by the LNN-GNDO-SQP algorithm using a fitness function as in Equation (54). Here three cases are studied by varying ϕ in the drainage problem.

	Case I			Case II			Case III		
Index	$\alpha_{j}$	$\omega_{j}$	$\beta_{j}$	$\alpha_{j}$	$\omega_{j}$	$\beta_{j}$	$\alpha_{j}$	$\omega_{j}$	$\beta_{j}$
1	−0.578170	0.090627	0.621742	−0.719570	0.999994	−0.664540	−0.841870	0.915168	−0.247580
2	0.642790	−0.552250	0.999977	0.863347	−0.300220	0.348017	0.456016	0.271674	0.687734
3	−0.984330	0.465779	−0.162840	−0.759410	−0.863760	0.994778	0.408307	−0.370890	0.016365
4	−0.620110	0.128942	0.566053	−0.413700	0.378054	0.137548	0.125005	−0.162090	−0.313500
5	0.924819	−0.630340	0.993488	−0.284960	−0.285900	0.692566	−0.316870	0.173333	0.750680
6	−0.282300	0.331916	0.220188	−0.354880	−0.672930	−0.554160	−0.118880	0.196454	−0.250040
7	0.999998	−0.258550	−0.093070	−0.000860	0.398419	−0.803340	0.886842	0.542877	−0.637760
8	−0.934180	0.280345	−0.632150	0.218599	−0.235300	−0.046750	−0.225580	−0.157780	−0.417790
9	−0.179500	−0.705000	0.207103	−0.460840	−0.145630	0.673652	0.425031	0.174465	0.278200
10	0.000931	0.269963	0.264995	−0.163930	0.280319	−0.353570	−0.998280	−0.306190	0.186654
11	0.367440	0.270983	−0.105050	0.161173	0.188646	−0.083490	−0.547840	0.271897	0.005882

**Table 10:** Best set of weights obtained by the LNN-GNDO-SQP algorithm using a fitness function as in Equation (55). Here three cases are studied by varying *a* in the drainage problem.

	Case I			Case II			Case III		
Index	$\alpha_{j}$	$\omega_{j}$	$\beta_{j}$	$\alpha_{j}$	$\omega_{j}$	$\beta_{j}$	$\alpha_{j}$	$\omega_{j}$	$\beta_{j}$
1	−0.937800	0.260055	−0.599770	−0.996410	0.505919	−0.254550	−1.357140	1.301247	−1.342750
2	0.373960	−0.384460	−0.286000	0.308871	0.389527	0.441955	1.620159	−0.810600	−0.543930
3	0.862114	0.210662	−0.163780	0.841584	0.082865	0.640732	0.153404	−1.187130	−0.400690
4	−0.996400	0.758110	−0.974980	0.992297	−0.456590	−0.13996	0.025061	0.244749	0.341770
5	−0.616540	−0.416200	0.113885	−0.844170	0.661252	−0.369140	−1.162260	−0.289160	−0.150160
6	−0.729390	0.223456	0.069204	0.348376	−0.289810	0.556567	−1.254670	−0.224680	−0.178620
7	0.810262	0.262491	−0.492080	−0.999670	−0.061340	−0.055630	1.221794	0.082876	0.282458
8	0.885861	−0.200570	−0.356040	0.256956	−0.775240	0.414591	0.300091	0.123791	0.165290
9	0.690933	0.109413	0.407307	0.054504	−0.465980	−0.080880	−1.119120	0.256081	0.135531
10	−0.702680	−0.316270	0.484328	0.289438	−0.128410	0.146051	0.830104	0.241196	0.291386
11	−0.080280	−0.212490	0.046098	−0.616560	0.295517	−1.000000	−1.171850	−0.266330	−0.063730

**Table 11:** Statistical analysis of the results obtained by the LNN-GNDO-SQP algorithm, GA-ASA technique on the 21 steps within the interval $\left[1\;2\right]$, It is evident that LNN-GNDO-SQP is superior in accuracy and stability. Three cases of drainage problem are considered based on variations in $S_{t}$.

	Case I						Case II						Case III					
	Min		Mean		Std		Min		Mean		Std		Min		Mean		Std	
	GA-ASA	GNDO-SQP	GA-ASA	GNDO-SQP	GA-ASA	GNDO-SQP	GA-ASA	GNDO-SQP	GA-ASA	GNDO-SQP	GA-ASA	GNDO-SQP	GA-ASA	GNDO-SQP	GA-ASA	GNDO-SQP	GA-ASA	GNDO-SQP
1	$5.7\times 10^{-13}$	** $7.36\times 10^{-15}$ **	$5.0\times 10^{-09}$	** $7.72\times 10^{-10}$ **	$3.8\times 10^{-08}$	** $2.73\times 10^{-09}$ **	$4.0\times 10^{-12}$	** $1.46\times 10^{-13}$ **	$1.3\times 10^{-07}$	$1.90\times 10^{-08}$	$5.7\times 10^{-07}$	$5.22\times 10^{-08}$	$1.9\times 10^{-12}$	** $7.92\times 10^{-15}$ **	$2.7\times 10^{-07}$	** $2.58\times 10^{-08}$ **	$8.4\times 10^{-07}$	** $7.13\times 10^{-08}$ **
1.05	$1.1\times 10^{-08}$	** $5.74\times 10^{-12}$ **	$1.2\times 10^{-06}$	** $2.01\times 10^{-09}$ **	$7.6\times 10^{-07}$	** $4.24\times 10^{-09}$ **	$6.8\times 10^{-08}$	** $5.04\times 10^{-11}$ **	$2.4\times 10^{-06}$	** $5.48\times 10^{-08}$ **	$1.5\times 10^{-06}$	** $8.88\times 10^{-08}$ **	$2.0\times 10^{-09}$	** $2.36\times 10^{-11}$ **	$1.8\times 10^{-06}$	** $1.08\times 10^{-07}$ **	$1.4\times 10^{-06}$	** $1.65\times 10^{-07}$ **
1.1	$1.6\times 10^{-08}$	** $2.22\times 10^{-13}$ **	$3.8\times 10^{-06}$	** $2.76\times 10^{-09}$ **	$2.6\times 10^{-06}$	** $8.25\times 10^{-09}$ **	$1.7\times 10^{-07}$	** $4.19\times 10^{-12}$ **	$6.6\times 10^{-06}$	** $7.09\times 10^{-08}$ **	$3.9\times 10^{-06}$	** $1.60\times 10^{-07}$ **	$1.0\times 10^{-07}$	** $3.18\times 10^{-13}$ **	$4.9\times 10^{-06}$	** $1.14\times 10^{-07}$ **	$4.0\times 10^{-06}$	** $2.52\times 10^{-07}$ **
1.15	$3.0\times 10^{-08}$	** $5.15\times 10^{-15}$ **	$5.5\times 10^{-06}$	** $1.19\times 10^{-09}$ **	$3.9\times 10^{-06}$	** $4.56\times 10^{-09}$ **	$1.7\times 10^{-07}$	** $2.08\times 10^{-17}$ **	$9.0\times 10^{-06}$	** $2.88\times 10^{-08}$ **	$5.4\times 10^{-06}$	** $8.43\times 10^{-08}$ **	$1.2\times 10^{-08}$	** $2.75\times 10^{-14}$ **	$6.4\times 10^{-06}$	** $3.53\times 10^{-08}$ **	$5.6\times 10^{-06}$	** $1.15\times 10^{-07}$ **
1.2	$1.0\times 10^{-08}$	** $9.24\times 10^{-13}$ **	$5.7\times 10^{-06}$	** $2.52\times 10^{-10}$ **	$4.4\times 10^{-06}$	** $5.98\times 10^{-10}$ **	$1.1\times 10^{-07}$	** $7.62\times 10^{-16}$ **	$8.7\times 10^{-06}$	** $6.22\times 10^{-09}$ **	$5.5\times 10^{-06}$	** $1.04\times 10^{-08}$ **	$2.9\times 10^{-09}$	** $1.77\times 10^{-12}$ **	$5.9\times 10^{-06}$	** $1.04\times 10^{-08}$ **	$5.6\times 10^{-06}$	** $1.13\times 10^{-08}$ **
1.25	$2.1\times 10^{-08}$	** $2.96\times 10^{-16}$ **	$4.7\times 10^{-06}$	** $4.58\times 10^{-10}$ **	$4.0\times 10^{-06}$	** $5.55\times 10^{-10}$ **	$5.5\times 10^{-08}$	** $2.20\times 10^{-13}$ **	$6.5\times 10^{-06}$	** $1.23\times 10^{-08}$ **	$4.3\times 10^{-06}$	** $1.47\times 10^{-08}$ **	$1.1\times 10^{-09}$	** $6.32\times 10^{-14}$ **	$4.1\times 10^{-06}$	** $2.91\times 10^{-08}$ **	$4.1\times 10^{-06}$	** $3.52\times 10^{-08}$ **
1.3	$4.5\times 10^{-08}$	** $2.74\times 10^{-17}$ **	$3.1\times 10^{-06}$	** $9.68\times 10^{-10}$ **	$3.2\times 10^{-06}$	** $2.47\times 10^{-09}$ **	$2.4\times 10^{-08}$	** $6.00\times 10^{-16}$ **	$3.6\times 10^{-06}$	** $2.43\times 10^{-08}$ **	$2.6\times 10^{-06}$	** $4.66\times 10^{-08}$ **	$1.2\times 10^{-08}$	** $5.12\times 10^{-13}$ **	$2.0\times 10^{-06}$	** $4.42\times 10^{-08}$ **	$2.1\times 10^{-06}$	** $8.52\times 10^{-08}$ **
1.35	$2.8\times 10^{-09}$	** $1.27\times 10^{-13}$ **	$1.6\times 10^{-06}$	** $1.13\times 10^{-09}$ **	$2.2\times 10^{-06}$	** $3.96\times 10^{-09}$ **	$1.7\times 10^{-09}$	** $1.15\times 10^{-14}$ **	$1.1\times 10^{-06}$	** $2.69\times 10^{-08}$ **	$1.4\times 10^{-06}$	** $7.11\times 10^{-08}$ **	$1.3\times 10^{-08}$	** $1.29\times 10^{-12}$ **	$8.0\times 10^{-07}$	** $3.97\times 10^{-08}$ **	$1.1\times 10^{-06}$	** $1.17\times 10^{-07}$ **
1.4	$6.9\times 10^{-10}$	** $1.46\times 10^{-14}$ **	$7.1\times 10^{-07}$	** $8.67\times 10^{-10}$ **	$1.4\times 10^{-06}$	** $3.39\times 10^{-09}$ **	$5.7\times 10^{-09}$	** $8.39\times 10^{-13}$ **	$1.2\times 10^{-06}$	** $2.01\times 10^{-08}$ **	$1.1\times 10^{-06}$	** $5.99\times 10^{-08}$ **	$1.3\times 10^{-08}$	** $3.22\times 10^{-14}$ **	$1.6\times 10^{-06}$	** $2.57\times 10^{-08}$ **	$1.4\times 10^{-06}$	** $8.77\times 10^{-08}$ **
1.45	$1.4\times 10^{-08}$	** $1.57\times 10^{-13}$ **	$6.4\times 10^{-07}$	** $4.80\times 10^{-10}$ **	$8.0\times 10^{-07}$	** $1.50\times 10^{-09}$ **	$8.3\times 10^{-09}$	** $1.73\times 10^{-12}$ **	$1.4\times 10^{-06}$	** $1.13\times 10^{-08}$ **	$1.4\times 10^{-06}$	** $2.61\times 10^{-08}$ **	$4.1\times 10^{-08}$	** $3.36\times 10^{-15}$ **	$1.7\times 10^{-06}$	** $1.65\times 10^{-08}$ **	$1.5\times 10^{-06}$	** $3.38\times 10^{-08}$ **
1.5	$2.2\times 10^{-09}$	** $2.32\times 10^{-17}$ **	$6.4\times 10^{-07}$	** $2.58\times 10^{-10}$ **	$9.0\times 10^{-07}$	** $3.57\times 10^{-10}$ **	$1.2\times 10^{-09}$	** $3.64\times 10^{-15}$ **	$1.0\times 10^{-06}$	** $6.80\times 10^{-09}$ **	$1.8\times 10^{-06}$	** $9.65\times 10^{-09}$ **	$8.4\times 10^{-09}$	** $4.54\times 10^{-14}$ **	$1.3\times 10^{-06}$	** $1.52\times 10^{-08}$ **	$1.5\times 10^{-06}$	** $2.22\times 10^{-08}$ **
1.55	$3.8\times 10^{-08}$	** $3.18\times 10^{-13}$ **	$1.8\times 10^{-06}$	** $3.25\times 10^{-10}$ **	$1.4\times 10^{-06}$	** $4.87\times 10^{-10}$ **	$1.3\times 10^{-07}$	** $3.50\times 10^{-12}$ **	$3.2\times 10^{-06}$	** $8.48\times 10^{-09}$ **	$2.3\times 10^{-06}$	** $1.14\times 10^{-08}$ **	$3.3\times 10^{-08}$	** $2.49\times 10^{-13}$ **	$2.7\times 10^{-06}$	** $1.75\times 10^{-08}$ **	$2.0\times 10^{-06}$	** $2.40\times 10^{-08}$ **
1.6	$5.6\times 10^{-08}$	** $1.10\times 10^{-16}$ **	$3.2\times 10^{-06}$	** $5.95\times 10^{-10}$ **	$2.2\times 10^{-06}$	** $1.98\times 10^{-09}$ **	$6.6\times 10^{-08}$	** $4.75\times 10^{-14}$ **	$5.9\times 10^{-06}$	** $1.38\times 10^{-08}$ **	$3.6\times 10^{-06}$	** $3.38\times 10^{-08}$ **	$3.1\times 10^{-08}$	** $5.64\times 10^{-14}$ **	$4.7\times 10^{-06}$	** $2.14\times 10^{-08}$ **	$3.8\times 10^{-06}$	** $5.59\times 10^{-08}$ **
1.65	$1.9\times 10^{-08}$	** $4.89\times 10^{-14}$ **	$4.5\times 10^{-06}$	** $8.48\times 10^{-10}$ **	$3.2\times 10^{-06}$	** $3.19\times 10^{-09}$ **	$6.3\times 10^{-08}$	** $2.24\times 10^{-12}$ **	$8.2\times 10^{-06}$	** $1.87\times 10^{-08}$ **	$5.2\times 10^{-06}$	** $5.42\times 10^{-08}$ **	$1.6\times 10^{-10}$	** $1.05\times 10^{-12}$ **	$6.3\times 10^{-06}$	** $2.50\times 10^{-08}$ **	$5.8\times 10^{-06}$	** $8.37\times 10^{-08}$ **
1.7	$1.1\times 10^{-07}$	** $5.40\times 10^{-16}$ **	$5.3\times 10^{-06}$	** $8.23\times 10^{-10}$ **	$3.9\times 10^{-06}$	** $2.71\times 10^{-09}$ **	$3.3\times 10^{-09}$	** $1.42\times 10^{-17}$ **	$9.6\times 10^{-06}$	** $1.84\times 10^{-08}$ **	$6.3\times 10^{-06}$	** $4.62\times 10^{-08}$ **	$1.6\times 10^{-08}$	** $2.83\times 10^{-16}$ **	$7.2\times 10^{-06}$	** $2.65\times 10^{-08}$ **	$7.0\times 10^{-06}$	** $6.74\times 10^{-08}$ **
1.75	$1.6\times 10^{-07}$	** $3.07\times 10^{-14}$ **	$5.5\times 10^{-06}$	** $4.88\times 10^{-10}$ **	$4.0\times 10^{-06}$	** $1.05\times 10^{-09}$ **	$1.3\times 10^{-08}$	** $2.94\times 10^{-12}$ **	$9.7\times 10^{-06}$	** $1.21\times 10^{-08}$ **	$6.5\times 10^{-06}$	** $1.92\times 10^{-08}$ **	$2.4\times 10^{-09}$	** $1.92\times 10^{-14}$ **	$7.2\times 10^{-06}$	** $2.14\times 10^{-08}$ **	$7.2\times 10^{-06}$	** $3.10\times 10^{-08}$ **
1.8	$3.0\times 10^{-08}$	** $7.97\times 10^{-14}$ **	$5.0\times 10^{-06}$	** $1.65\times 10^{-10}$ **	$3.6\times 10^{-06}$	** $2.47\times 10^{-10}$ **	$3.6\times 10^{-09}$	** $5.84\times 10^{-12}$ **	$8.3\times 10^{-06}$	** $4.86\times 10^{-09}$ **	$5.6\times 10^{-06}$	** $7.95\times 10^{-09}$ **	$9.1\times 10^{-08}$	** $7.91\times 10^{-13}$ **	$6.0\times 10^{-06}$	** $1.07\times 10^{-08}$ **	$6.0\times 10^{-06}$	** $1.66\times 10^{-08}$ **
1.85	$2.8\times 10^{-08}$	** $1.28\times 10^{-14}$ **	$3.8\times 10^{-06}$	** $4.91\times 10^{-10}$ **	$3.1\times 10^{-06}$	** $1.74\times 10^{-09}$ **	$1.9\times 10^{-09}$	** $1.06\times 10^{-13}$ **	$5.7\times 10^{-06}$	** $1.01\times 10^{-08}$ **	$3.8\times 10^{-06}$	** $2.84\times 10^{-08}$ **	$3.9\times 10^{-09}$	** $1.81\times 10^{-14}$ **	$3.9\times 10^{-06}$	** $1.44\times 10^{-08}$ **	$3.9\times 10^{-06}$	** $4.67\times 10^{-08}$ **
1.9	$4.7\times 10^{-08}$	** $6.64\times 10^{-14}$ **	$2.4\times 10^{-06}$	** $1.45\times 10^{-09}$ **	$3.1\times 10^{-06}$	** $4.59\times 10^{-09}$ **	$7.2\times 10^{-08}$	** $2.93\times 10^{-12}$ **	$2.7\times 10^{-06}$	** $3.12\times 10^{-08}$ **	$2.0\times 10^{-06}$	** $7.28\times 10^{-08}$ **	$3.8\times 10^{-09}$	** $1.88\times 10^{-13}$ **	$1.9\times 10^{-06}$	** $4.69\times 10^{-08}$ **	$1.6\times 10^{-06}$	** $1.13\times 10^{-07}$ **
1.95	$3.1\times 10^{-08}$	** $1.31\times 10^{-12}$ **	$1.4\times 10^{-06}$	** $1.33\times 10^{-09}$ **	$3.3\times 10^{-06}$	** $3.11\times 10^{-09}$ **	$2.8\times 10^{-09}$	** $1.73\times 10^{-11}$ **	$1.3\times 10^{-06}$	** $3.14\times 10^{-08}$ **	$2.0\times 10^{-06}$	** $5.16\times 10^{-08}$ **	$1.3\times 10^{-08}$	** $2.77\times 10^{-12}$ **	$1.7\times 10^{-06}$	** $5.29\times 10^{-08}$ **	$1.8\times 10^{-06}$	** $8.46\times 10^{-08}$ **
2	$3.5\times 10^{-08}$	** $1.44\times 10^{-15}$ **	$1.3\times 10^{-06}$	** $2.64\times 10^{-10}$ **	$2.7\times 10^{-06}$	** $9.19\times 10^{-10}$ **	$2.8\times 10^{-09}$	** $7.27\times 10^{-14}$ **	$1.2\times 10^{-06}$	** $5.42\times 10^{-09}$ **	$2.1\times 10^{-06}$	** $1.43\times 10^{-08}$ **	$2.1\times 10^{-08}$	** $3.73\times 10^{-15}$ **	$1.6\times 10^{-06}$	** $7.45\times 10^{-09}$ **	$1.9\times 10^{-06}$	** $2.13\times 10^{-08}$ **

**Table 12:** Fitness values, MAD, TIC RMSE and ENSE for SCENARIO I of the drainage problem.

		Fit			MAD			TIC			RMSE			ENSE	
Cases	Min.	Mean	Std.	Min.	Mean	Std.	Min.	Mean	Std.	Min.	Mean	Std.	Min.	Mean	Std.
I	$1.49\times 10^{-12}$	$1.63\times 10^{-09}$	$4.68\times 10^{-09}$	$4.99\times 10^{-08}$	$1.84\times 10^{-06}$	$2.44\times 10^{-06}$	$2.49\times 10^{-07}$	$9.04\times 10^{-06}$	$1.23\times 10^{-05}$	$6.13\times 10^{-08}$	$2.22\times 10^{-06}$	$3.03\times 10^{-06}$	$1.36\times 10^{-10}$	$5.07\times 10^{-07}$	$1.46\times 10^{-06}$
II	$1.20\times 10^{-11}$	$3.96\times 10^{-08}$	$8.33\times 10^{-08}$	$1.70\times 10^{-07}$	$8.94\times 10^{-06}$	$1.05\times 10^{-05}$	$2.51\times 10^{-07}$	$1.47\times 10^{-05}$	$1.76\times 10^{-05}$	$1.85\times 10^{-07}$	$1.09\times 10^{-05}$	$1.30\times 10^{-05}$	$1.76\times 10^{-10}$	$1.15\times 10^{-06}$	$2.59\times 10^{-06}$
III	$4.51\times 10^{-12}$	$6.43\times 10^{-08}$	$1.30\times 10^{-07}$	$2.12\times 10^{-07}$	$1.08\times 10^{-05}$	$1.12\times 10^{-05}$	$2.59\times 10^{-07}$	$1.35\times 10^{-05}$	$1.42\times 10^{-05}$	$2.55\times 10^{-07}$	$1.33\times 10^{-05}$	$1.40\times 10^{-05}$	$1.54\times 10^{-10}$	$8.26\times 10^{-07}$	$1.72\times 10^{-06}$

**Table 13:** Fitness values, MAD, TIC RMSE and ENSE for SCENARIO II of the drainage problem.

		Fit			MAD			TIC			RMSE			ENSE	
Cases	Min.	Mean	Std.	Min.	Mean	Std.	Min.	Mean	Std.	Min.	Mean	Std.	Min.	Mean	Std.
I	$4.34\times 10^{-11}$	$1.18\times 10^{-07}$	$2.35\times 10^{-07}$	$1.86\times 10^{-07}$	$1.87\times 10^{-05}$	$1.98\times 10^{-05}$	$1.86\times 10^{-07}$	$1.81\times 10^{-05}$	$1.96\times 10^{-05}$	$2.29\times 10^{-07}$	$2.22\times 10^{-05}$	$2.41\times 10^{-05}$	$7.57\times 10^{-11}$	$1.61\times 10^{-06}$	$3.32\times 10^{-06}$
II	$1.05\times 10^{-10}$	$2.78\times 10^{-07}$	$6.38\times 10^{-07}$	$2.67\times 10^{-07}$	$2.28\times 10^{-05}$	$2.58\times 10^{-05}$	$2.59\times 10^{-07}$	$2.18\times 10^{-05}$	$2.53\times 10^{-05}$	$3.31\times 10^{-07}$	$2.79\times 10^{-05}$	$3.24\times 10^{-05}$	$1.47\times 10^{-10}$	$2.43\times 10^{-06}$	$5.93\times 10^{-06}$
III	$1.50\times 10^{-09}$	$3.21\times 10^{-07}$	$6.88\times 10^{-07}$	$3.51\times 10^{-07}$	$1.66\times 10^{-05}$	$1.76\times 10^{-05}$	$3.10\times 10^{-07}$	$1.49\times 10^{-05}$	$1.55\times 10^{-05}$	$4.23\times 10^{-07}$	$2.03\times 10^{-05}$	$2.11\times 10^{-05}$	$2.29\times 10^{-10}$	$1.08\times 10^{-06}$	$2.60\times 10^{-06}$

**Table 14:** Fitness values, MAD, TIC RMSE and ENSE for SCENARIO III of the drainage problem.

		Fit			MAD			TIC			RMSE			ENSE	
Cases	Min.	Mean	Std.	Min.	Mean	Std.	Min.	Mean	Std.	Min.	Mean	Std.	Min.	Mean	Std.
I	$6.63\times 10^{-12}$	$6.37\times 10^{-08}$	$1.48\times 10^{-07}$	$3.67\times 10^{-07}$	$1.45\times 10^{-05}$	$1.53\times 10^{-05}$	$3.54\times 10^{-07}$	$1.46\times 10^{-05}$	$1.57\times 10^{-05}$	$4.35\times 10^{-07}$	$1.79\times 10^{-05}$	$1.93\times 10^{-05}$	$2.95\times 10^{-10}$	$9.69\times 10^{-07}$	$2.16\times 10^{-06}$
II	$1.08\times 10^{-12}$	$1.42\times 10^{-08}$	$4.90\times 10^{-08}$	$1.76\times 10^{-07}$	$4.45\times 10^{-06}$	$6.15\times 10^{-06}$	$1.93\times 10^{-07}$	$5.09\times 10^{-06}$	$7.07\times 10^{-06}$	$2.10\times 10^{-07}$	$5.53\times 10^{-06}$	$7.68\times 10^{-06}$	$8.21\times 10^{-11}$	$1.52\times 10^{-07}$	$5.00\times 10^{-07}$
III	$1.08\times 10^{-12}$	$9.66\times 10^{-09}$	$2.72\times 10^{-08}$	$2.82\times 10^{-07}$	$3.52\times 10^{-06}$	$5.31\times 10^{-06}$	$3.51\times 10^{-07}$	$4.30\times 10^{-06}$	$6.37\times 10^{-06}$	$3.53\times 10^{-07}$	$4.32\times 10^{-06}$	$6.40\times 10^{-06}$	$2.39\times 10^{-10}$	$1.21\times 10^{-07}$	$3.61\times 10^{-07}$

**Table 15:** Fitness values, MAD, TIC RMSE and ENSE for SCENARIO IV of the drainage problem.

		Fit			MAD			TIC			RMSE			ENSE	
Cases	Min.	Mean	Std.	Min.	Mean	Std.	Min.	Mean	Std.	Min.	Mean	Std.	Min.	Mean	Std.
I	$7.66\times 10^{-11}$	$1.95\times 10^{-07}$	$3.96\times 10^{-07}$	$2.25\times 10^{-06}$	$3.62\times 10^{-05}$	$3.49\times 10^{-05}$	$1.43\times 10^{-06}$	$2.49\times 10^{-05}$	$2.45\times 10^{-05}$	$2.47\times 10^{-06}$	$4.29\times 10^{-05}$	$4.23\times 10^{-05}$	$5.63\times 10^{-09}$	$2.81\times 10^{-06}$	$5.56\times 10^{-06}$
II	$3.20\times 10^{-12}$	$1.47\times 10^{-06}$	$3.08\times 10^{-06}$	$3.17\times 10^{-06}$	$8.21\times 10^{-05}$	$8.21\times 10^{-05}$	$1.99\times 10^{-06}$	$5.13\times 10^{-05}$	$5.26\times 10^{-05}$	$3.90\times 10^{-06}$	0.000101	0.000103	$9.23\times 10^{-09}$	$1.23\times 10^{-05}$	$2.57\times 10^{-05}$
III	$5.00\times 10^{-09}$	$1.25\times 10^{-06}$	$2.93\times 10^{-06}$	$4.37\times 10^{-06}$	$4.59\times 10^{-05}$	$6.62\times 10^{-05}$	$2.31\times 10^{-06}$	$2.48\times 10^{-05}$	$3.57\times 10^{-05}$	$4.99\times 10^{-06}$	$5.36\times 10^{-05}$	$7.72\times 10^{-05}$	$1.50\times 10^{-08}$	$5.05\times 10^{-06}$	$1.17\times 10^{-05}$

## Data Availability

The data that support the findings of this study are available from the corresponding author upon reasonable request.

## Abbreviations

LNN	Legendre Neural Networks
GNDO	Generalized Normal Distribution Optimization
SQP	Sequential Quadratic Programming
ANN	Artificial Neural Networks
MAD	Mean Absolute Deviation
TIC	Theil’s inequality coefficient
NSE	Nash Sutcliffe Efficiency
ENSE	Error In Nash Sutcliffe Efficiency
RMSE	Root Mean Square Error
MHD	Magnetohydrodynamic
$W_{e}$	Weissenberg number
*M*	Mean Position
$S_{t}$	Stokes Number
*a*	Slip Parameter
ϕ	Ratio of Viscosities
$\underline{V}$	Velocity Vector
ρ	Density
σ	Cauchy Stress Tensor
μ,η	Viscosities
$u^{t}$	Trial vector
δ	Thinkness of Thin Film
$\mu_{i}$	generalized Mean
$\delta_{i}$	Generalized Variance
β	Adjustment Parameter

## Appendix A

Approximate series solutions obtained by LNN-GNDO-SQP algorithm for different cases of scenario I.

Case I

(A1) $$\begin{array}{cc} & w(r)=-0.267240+(0.083281r+0.361633)(0.199856)+\cdots \\ & +\left(\frac{90090{\left(-0.225430r+0.133951\right)}^{6}-30030{\left(-0.225430r+0.133951\right)}^{4}}{256}\right)\left(-0.362530\right)\\ & +\left(\frac{3465{\left(-0.225430r+0.133951\right)}^{2}-63}{256}\right)\left(-0.362530\right) \end{array}$$

Case II

(A2) $$\begin{array}{cc} & u_{approx}\left(\xi \right)=-0.161130+\left(-0.082950r-0.133580\right)\left(0.540048\right)+\cdots \\ & +\left(\frac{90090{\left(0.041990r+0.288770\right)}^{6}-30030{\left(0.041990r+0.288770\right)}^{4}}{256}\right)\left(0.999719\right)\\ & +\left(\frac{3465{\left(0.041990r+0.288770\right)}^{2}-63}{256}\right)\left(0.999719\right) \end{array}$$

Case III

(A3) $$\begin{array}{cc} & u_{approx}\left(\xi \right)=-0.642690+\left(-0.085350r+0.282074\right)\left(-0.500960\right)+\cdots \\ & +\left(\frac{90090{\left(-0.229960r-0.050250\right)}^{6}-30030{\left(-0.229960r-0.050250\right)}^{4}}{256}\right)\left(-0.148830\right)\\ & +\left(\frac{3465{\left(-0.229960r-0.050250\right)}^{2}-63}{256}\right)\left(-0.148830\right) \end{array}$$

Approximate series solutions obtained by LNN-GNDO-SQP algorithm for different cases of scenario II.

Case I

(A4) $$\begin{array}{cc} & w(r)=-0.277180+(0.9900770r-0.999960)(-0.744240)+\cdots \\ & +\left(\frac{90090{\left(-0.744240r+0.101130\right)}^{6}-30030{\left(-0.744240r+0.101130\right)}^{4}}{256}\right)\left(-0.629380\right)\\ & +\left(\frac{3465{\left(-0.744240r+0.101130\right)}^{2}-63}{256}\right)\left(-0.629380\right) \end{array}$$

Case II

(A5) $$\begin{array}{cc} & u_{approx}\left(\xi \right)=-0.984680+\left(0.555550r-0.024790\right)\left(-0.801200\right)+\cdots \\ & +\left(\frac{90090{\left(-0.138270r+0.103016\right)}^{6}-30030{\left(-0.138270r+0.103016\right)}^{4}}{256}\right)\left(-0.138360\right)\\ & +\left(\frac{3465{\left(-0.138270r+0.103016\right)}^{2}-63}{256}\right)\left(-0.138360\right) \end{array}$$

Case III

(A6) $$\begin{array}{cc} & u_{approx}\left(\xi \right)=-0.575380+\left(0.131423r+0.343622\right)\left(-0.999990\right)+\cdots \\ & +\left(\frac{90090{\left(0.174965r+0.126845\right)}^{6}-30030{\left(0.174965r+0.126845\right)}^{4}}{256}\right)\left(-0.148830\right)\\ & +\left(\frac{3465{\left(0.174965r+0.126845\right)}^{2}-63}{256}\right)\left(-0.148830\right) \end{array}$$

Approximate series solutions obtained by LNN-GNDO-SQP algorithm for different cases of scenario III.

Case I

(A7) $$\begin{array}{cc} & u_{approx}\left(\xi \right)=-0.578170+\left(-0.552250r+0.999977\right)\left(0.64279\right)+\cdots \\ & +\left(\frac{90090{\left(0.270983r-0.10505\right)}^{6}-30030{\left(0.270983r-0.10505\right)}^{4}}{256}\right)\left(-0.148830\right)\\ & +\left(\frac{3465{\left(0.270983r-0.10505\right)}^{2}-63}{256}\right)\left(-0.148830\right) \end{array}$$

Case II

(A8) $$\begin{array}{cc} & u_{approx}\left(\xi \right)=-0.719570+\left(-0.300220r+0.348017\right)\left(0.863347\right)+\cdots \\ & +\left(\frac{90090{\left(0.188646r-0.08349\right)}^{6}-30030{\left(0.188646r-0.08349\right)}^{4}}{256}\right)\left(0.161173\right)\\ & +\left(\frac{3465{\left(0.188646r-0.08349\right)}^{2}-63}{256}\right)\left(0.161173\right) \end{array}$$

Case III

(A9) $$\begin{array}{cc} & u_{approx}\left(\xi \right)=-0.84187+\left(0.271674r+0.687734\right)\left(0.456016\right)+\cdots \\ & +\left(\frac{90090{\left(0.271897r+0.005882\right)}^{6}-30030{\left(0.271897r+0.005882\right)}^{4}}{256}\right)\left(-0.547840\right)\\ & +\left(\frac{3465{\left(0.271897r+0.005882\right)}^{2}-63}{256}\right)\left(-0.547840\right) \end{array}$$

Approximate series solutions obtained by LNN-GNDO-SQP algorithm for different cases of scenario IV.

Case I

(A10) $$\begin{array}{cc} & u_{approx}\left(\xi \right)=-0.937800+\left(-0.384460r-0.286000\right)\left(0.373960\right)+\cdots \\ & +\left(\frac{90090{\left(-0.212490r+0.046098\right)}^{6}-30030{\left(-0.212490r+0.046098\right)}^{4}}{256}\right)\left(-0.080280\right)\\ & +\left(\frac{3465{\left(-0.212490r+0.046098\right)}^{2}-63}{256}\right)\left(-0.080280\right) \end{array}$$

Case II

(A11) $$\begin{array}{cc} & u_{approx}\left(\xi \right)=-0.996410+\left(0.389527r+0.441955\right)\left(0.308871\right)+\cdots \\ & +\left(\frac{90090{\left(0.295517r-1.000000\right)}^{6}-30030{\left(0.295517r-1.000000\right)}^{4}}{256}\right)\left(-0.616560\right)\\ & +\left(\frac{3465{\left(0.295517r-1.000000\right)}^{2}-63}{256}\right)\left(-0.616560\right) \end{array}$$

Case III

(A12) $$\begin{array}{cc} & u_{approx}\left(\xi \right)=-1.357140+\left(-0.810600r-0.543930\right)\left(1.620159\right)+\cdots \\ & +\left(\frac{90090{\left(-0.266330r-0.063730\right)}^{6}-30030{\left(-0.266330r-0.063730\right)}^{4}}{256}\right)\left(-1.171850\right)\\ & +\left(\frac{3465{\left(-0.266330r-0.063730\right)}^{2}-63}{256}\right)\left(-1.171850\right) \end{array}$$
